# Sustained-release enteric formulations of *Lactiplantibacillus plantarum* based on granulation technology: preparation and therapeutic evaluation in acute colitis

**DOI:** 10.1186/s12896-026-01157-7

**Published:** 2026-04-21

**Authors:** Xiwei Zhang, Jianfeng Wan, Xile Wang, Xiaocan Zhang, Miaomiao Jiang, Shuang Liang, Hongqing Zhang, Guangzhou Zhou

**Affiliations:** 1 College of Biological Engineering, Henan University of Technology, No. 100 Lianhua Street, Zhengzhou, 450052 China; 2 Zhengzhou Qinghuayuan Bioengineering Co. Ltd, No. 5 Hehuan Street, Zhengzhou, 450052 China

**Keywords:** Acute colitis, *Lactiplantibacillus plantarum*, Enteric sustained-release pellets, HPMCP, Eudragit L100

## Abstract

**Background:**

The clinical effectiveness of orally administered probiotics is often limited by poor survival during gastrointestinal transit and insufficient delivery to the intestinal site of action. *Lactiplantibacillus plantarum*, a probiotic with anti-inflammatory potential, is highly sensitive to gastric acid and bile salts, diminishing its therapeutic efficacy for intestinal diseases. The aim of this study was to develop an enteric sustained-release granule formulation of Lactobacillus plantarum to enhance probiotic stability, promote targeted intestinal delivery, and evaluate its therapeutic efficacy in the treatment of colitis.

**Methods:**

Sustained-release enteric pellets containing *Lactiplantibacillus plantarum* were prepared using a low-temperature wet granulation and extrusion process, followed by coating with pH-responsive polymers (HPMCP or Eudragit L100). In vitro release behavior was evaluated in simulated gastric and intestinal fluids. Therapeutic efficacy was assessed in a dextran sulfate sodium (DSS)-induced acute colitis mouse model using histopathology, macrophage polarization, and gut microbiota analysis.

**Results:**

The sustained-release granules exhibited a narrow and uniform particle size distribution, with diameters ranging from 40 to 80 mesh (180–425 μm). More than 85% of the granules were distributed within the 40–60 mesh fraction (250–425 μm). The micropellets exhibited adequate mechanical strength for handling and processing. The enteric-coated micropellets showed pronounced acid resistance, retaining over 70% bacterial viability after 2 h in simulated gastric fluid, compared with <30% for free bacterial powder. In simulated intestinal fluid, the micropellets achieved sustained bacterial release for over 2 h. In DSS-induced colitis mice, treatment with the granule formulations resulted in a more pronounced trend toward histological recovery. Moreover, the sustained-release granules promoted M2 macrophage polarization and partially restored gut microbial diversity, as evidenced by normalization of the Firmicutes-to-Bacteroidetes ratio.

**Conclusions:**

The sustained-release enteric pellet system effectively protects *Lactiplantibacillus plantarum* during gastrointestinal transit and achieves targeted intestinal release. Integrating mechanical shielding with pH-triggered dissolution significantly enhances probiotic gastrointestinal survival and therapeutic efficacy. This delivery platform represents a safe, scalable, and broadly applicable strategy for treating intestinal inflammatory diseases. Furthermore, the approach provides a versatile framework for delivering other probiotic strains or combinations.

**Supplementary Information:**

The online version contains supplementary material available at 10.1186/s12896-026-01157-7.

## Background

Probiotics, particularly *Lactiplantibacillus plantarum*(formerly *Lactobacillus plantarum*), have demonstrated therapeutic potential in regulating gut microbiota and promoting intestinal health [1, 2]. However, a major challenge in probiotic-based therapy for intestinal diseases is ensuring the survival and targeted delivery of orally administered probiotics. These beneficial bacteria are exposed to harsh gastrointestinal conditions, including gastric acid, bile salts, and digestive enzymes, which drastically reduce their viability and limit clinical efficacy [3]. Conventional formulations such as fast-release powders or capsules provide insufficient protection during gastrointestinal transit and lack targeted release mechanisms, thereby failing to ensure an adequate number of viable bacteria reach the colon [4].

In recent years, multi-unit pellet systems have emerged as a promising strategy for oral drug delivery [5, 6]. Composed of numerous small granules, these systems disperse uniformly throughout the gastrointestinal tract, reducing local irritation and minimizing the risk of dose dumping. Compared to conventional monolithic dosage forms, multi-unit systems offer the advantage of tailored release profiles, including sustained or delayed release, and can be engineered for site-specific delivery based on pH or transit time. For probiotic formulations, enteric polymer coatings have been employed to protect bacteria under acidic conditions while enabling their release in the intestine [7], thereby enhancing intestinal colonization and activity [8, 9].

Despite these advancements, current probiotic delivery technologies exhibit limitations in optimizing viability and targeted release. Rapid-release formulations may prematurely discharge probiotics in the stomach, leading to substantial bacterial loss, while standard capsule-based systems cannot achieve the uniform dispersion and precise release control afforded by multi-unit approaches.

Hydroxypropyl methylcellulose phthalate (HPMCP) and Eudragit L100 are among the most widely used pH-responsive enteric polymers for oral delivery of acid-labile compounds and probiotics. HPMCP, a cellulose-based enteric material, protects sensitive agents from gastric degradation by forming a hydrophobic and insoluble coating under acidic conditions (pH 1–3), where its carboxyl groups remain protonated and form dense intermolecular hydrogen-bond networks that effectively block water penetration. When the environmental pH increases to its dissolution threshold (typically pH 5.0–5.8), ionization of carboxyl groups disrupts these hydrogen bonds, leading to polymer relaxation and dissolution, thus enabling site-specific release within the intestinal tract [10, 11]. Similarly, Eudragit polymers, which are methacrylic acid and acrylic ester copolymers, exhibit anionically driven pH-dependent solubility. In acidic gastric media, undissociated carboxyl groups render Eudragit L and S insoluble, forming a robust protective barrier. Once the pH exceeds their respective dissolution thresholds (pH > 5.5 for L, pH > 7.0 for S), ionization generates carboxylate anions (–COO^−^), relaxing the polymer matrix and allowing controlled dissolution [7, 12]. Specifically, Eudragit L100, which dissolves around pH 6.0, can facilitate delayed or site-specific release from the distal small intestine to the colon [13].

Recent advances in pH-responsive polymer science have revitalized research interest in these classical materials, revealing new insights into their structure–function relationships and formulation versatility. In parallel, recent reviews have highlighted the rapid evolution of micro- and nanoparticulate gastrointestinal delivery systems, emphasizing the critical role of enteric polymers in achieving spatially controlled release and enhanced stability under harsh gastric conditions [14]. Multiscale structural analyses of gelatin/HPMCP composites demonstrated dynamic morphological transitions across pH 2–6, highlighting the tunable network behavior of HPMCP and its potential for precision release control [15].

In parallel, Eudragit L100 continues to attract attention due to its excellent gastric resistance and customizable dissolution threshold. Recent studies have extended its application in probiotic encapsulation and stabilization: Combining Eudragit L100 with trehalose during single-droplet drying has been shown to significantly enhance the viability of Lacticaseibacillus rhamnosus GG by modulating particle morphology and rehydration dynamics [16]; It has been demonstrated that L100–trehalose–polysaccharide microcapsules fabricated by spray drying can improve bacterial adhesion and acid tolerance [17]. Collectively, these studies illustrate that despite their long-standing use, both HPMCP and Eudragit L100 remain at the forefront of enteric polymer research owing to their chemical adaptability, process versatility, and potential for engineering next-generation oral delivery systems.

Therefore, the aim of this study was to design an enteric sustained-release granule system for *Lactiplantibacillus plantarum* and to evaluate its stability, controlled release behavior, and therapeutic efficacy in acute colitis, with particular emphasis on intestinal inflammation and microbiota modulation. The formulation employs a sucrose-based filler phase cross-linked with pH-responsive polymers to construct a release matrix capable of withstanding gastric conditions while enabling controlled release of viable bacteria in the intestinal environment. Through systematic material screening, in vitro release profiling, and in vivo evaluation using a murine model of acute colitis, the study aims to elucidate the release mechanism and therapeutic potential of the proposed delivery system. By enhancing site-specific efficacy, this strategy could reduce the per-dose probiotic requirement while maintaining or improving therapeutic outcomes, thereby alleviating patient treatment burden. Furthermore, this work provides valuable insights into the design and application of next-generation oral probiotic delivery systems and offers a viable pathway for addressing current limitations in probiotic therapy. Importantly, this technological approach offers a promising platform not only for single-strain applications but also for delivering multi-strain or composite probiotic formulations.

## Materials and methods

### Experimental materials

*Lactiplantibacillus plantarum* (viable count  ≥ 1 × 10^10^ CFU/g); Sodium alginate (SA), cellulose acetate phthalate (CAP) (Chemicell); hydroxypropyl methylcellulose succinate (HPMCAS), hydroxypropyl methylcellulose phthalate (HPMCP) (Wuhan Lanabai Pharmaceutical Chemical Co., Ltd.); Eudragit L100 and S100 (Shanghai Dexiang Pharmaceutical Technology Co., Ltd.).Dextran sulfate sodium salt (DSS) (Yisheng Biotechnology, Shanghai, China). Male BALB/c mice (6–8 weeks old, 25 g) were purchased from Zhengzhou University Experimental Animal Center. Electrically heated constant-temperature drying oven (Shanghai Shuli Instrument Co., Ltd.); autoclave sterilizer (Zhejiang Xinfeng Medical Devices Co., Ltd.); carbon dioxide incubator with temperature-controlled shaker; ultrapure water system (Ningbo Dansboton Environmental Technology Co., Ltd.).

### Probiotic cultures and plate counts

*Lactiplantibacillus plantarum* was cultured in de Man, Rogosa and Sharpe (MRS) broth at 37 °C under anaerobic conditions for 18–24 h until reaching the late exponential phase. Cells were harvested by centrifugation at 4,000 × g for 10 min, washed twice with sterile phosphate-buffered saline (PBS), and resuspended to the desired concentration. The viable cell count was determined by plate counting on MRS agar following incubation at 37 °C for 48 h.

### Screening of antibacterial properties of coating materials

The antibacterial activity of different coating materials was assessed using the inhibition zone method to identify suitable excipients for probiotic encapsulation. Thirty microliters of *L. plantarum* suspension (adjusted to ~ 1 × 10^8^ CFU/mL after MRS liquid culture) were evenly spread onto MRS agar plates. Circular sterile filter papers (6 mm diameter) were soaked in 2% (w/v) solutions of Sodium alginate (SA), hydroxypropyl methylcellulose phthalate (HPMCP), cellulose acetate phthalate (CAP), hydroxypropyl methylcellulose succinate (HPMCAS), Eudragit L100 (L100), and Eudragit S100 (S100), then placed onto the plates. Plates were incubated inverted at 37 ± 0.5 °C for 24 ± 2 hours. Filter papers soaked in antibiotic solution and sterile water served as positive and negative controls, respectively. The diameter of inhibition zones was measured; smaller zones indicated lower antibacterial activity. All experiments were performed in triplicate, and average values were recorded.

### Preparation and optimization of sustained-release granules

#### Granule preparation and process optimization

To avoid thermal inactivation of the probiotic during granule drying, low-temperature drying (35–40 °C) was employed, while other steps (crushing, sieving, mixing, extrusion) followed conventional protocols. For HPMCP-based granules, 1% (w/v) HPMCP solution (dissolved in a 1:1 methanol-acetone mixture) was gradually added to a mortar containing freeze-dried *L. plantarum* powder premixed with sucrose (1:1). The mixture was ground until a non-sticky, coarse, granular material formed. The mixture was sieved sequentially through 40-mesh and then 80-mesh sieves. Particles retained on the 80-mesh sieve (particle size range: 180–425 μm) were and dried at 37–40 °C for 22–26 hours.

Granules were similarly prepared using SA, HPMCP, CAP, HPMCAS, L100, and S100 at concentrations of 0.5, 1, and 2% (w/v). Solvents were sterile water for SA, methanol-acetone for CAP and HPMCAS, and 75% ethanol for L100 and S100.

#### Disintegration test in simulated gastric and intestinal fluids

The disintegration behavior of granules was evaluated in simulated gastric fluid (SGF) and simulated intestinal fluid (SIF) according to the United States Pharmacopeia (USP) guidelines, with minor modifications. Simulated gastric fluid (SGF, pH 1.2, without enzymes) was prepared by dissolving 2.0 g of sodium chloride in purified water, adding 7.0 mL of concentrated hydrochloric acid, and adjusting the final volume to 1 L. Simulated intestinal fluid (SIF, pH 6.8, without enzymes) was prepared by dissolving 6.8 g of potassium dihydrogen phosphate in purified water, adjusting the pH to 6.8 with sodium hydroxide solution, and making up the final volume to 1 L.

For the disintegration test, 10 mL of SGF was preheated to 37 ± 0.5 °C, and 0.5 g of the granule formulation was added. The samples were incubated in a shaking incubator at 150 rpm to simulate gastric conditions. The morphology and integrity of the granules were visually monitored over a period of 4 h to assess their acid resistance. 0.5 g of the granules was added to 10 mL of preheated SIF and incubated under the same conditions. The disintegration process and the time required for complete disintegration were recorded to evaluate intestinal disintegration behavior. Disintegration was defined as the complete loss of the original granule structure, with no intact particles remaining, consistent with USP criteria. The use of a shaking incubator was adopted as a minor modification to better simulate dynamic gastrointestinal conditions.

#### Dissolution - viable bacterial release in simulated gastric and intestinal fluids

The in vitro release and survival of viable *Lactiplantibacillus plantarum* from the granules were evaluated using a modified USP-based gastrointestinal simulation model. 0.5 g of granules was added to 10 mL of preheated SGF and incubated in a shaking incubator at 37 ± 0.5 °C and 150 rpm. At predetermined time points (0, 30, 60, 90 and 120 min), 0.5 mL aliquots were withdrawn, serially diluted with sterile PBS, and plated on MRS agar. Plates were incubated at 37 °C for 24–48 h, and colony-forming units (CFU) were counted to determine the survival rate of the bacteria under gastric conditions.

Subsequently, the granules were transferred to 10 mL of preheated SIF and incubated under the same conditions. Samples were collected at 0, 30, 60, 90 and 120 min, diluted, plated, and incubated as described above. Count the number of viable bacteria released at each time point and plot cumulative release curves.

#### Scanning electron microscopy (SEM)

The surface morphology of the granules was observed using scanning electron microscopy (SEM). The samples were sputter-coated with a thin layer of gold and examined under a scanning electron microscope (HITACHI SU8020) at an accelerating voltage of 5kV. SEM analysis was conducted on granules formulated with HPMCP and L100, selected based on their favorable dissolution and non-antibacterial properties.

### Induction of acute colitis and treatment protocol

After a 3-day acclimation period, mice were randomly assigned into five groups: Control group (*n* = 3), DSS model group (*n* = 3), L. plantarum group (*n* = 3), HPMCP granule group (*n* = 3), and L100 granule group (*n* = 3). During the modeling phase, all groups except the normal group received 3% (w/v) DSS in drinking water ad libitum for 7 days, with daily replacement of freshly prepared solutions to induce acute colitis mimicking human pathophysiology (Table 1).

**Table 1:** Experimental design for colitis induction and treatment

Stage	Group	Intervention
Modeling Phase	Normal group (*n* = 3)	Free access to drinking water (pure water)
	Colitis model group (*n* = 12)	Free access to 3% DSS solution in drinking water
Treatment Phase	Normal group (*n* = 3)	Sterile water
	DSS group(*n* = 3)	Sterile water
	*Lactiplantibacillus plantarum* group (*n* = 3)	*Lactiplantibacillus plantarum* powder
	HPMCP group (*n* = 3)	*HPMCP-L. plantarum* sustained-release granules
	L100 group (*n* = 3)	*L100-L. plantarum* sustained-release granules

Starting on Day 8, mice were treated via oral gavage every other day for a total of three doses (Days 8, 10, and 12). The dose administered was 100 mg per mouse and the vehicle for administration was sterile purified water. The dose volume was determined according to body weight. The standard oral gavage volume of mice was 0.1–0.2 mL/10 g body weight, and the maximum single dose did not exceed 0.4 mL. Granular or powder formulations are prepared as suspensions just prior to administration. Each suspension was vortexed thoroughly prior to gavage to ensure homogeneity and prevent settling of particles, thereby ensuring accurate dosing. The HPMCP and L100 groups received suspensions of respective granules; the powder group received equivalent doses of free *L. plantarum* powder; the DSS and normal groups received sterile water. All gavage procedures were conducted under sterile conditions.

On day 14, experimental mice were intraperitoneally administered 1% pentobarbital sodium solution (10 mg/mL) at a dose of 100 mg/kg to induce a stable state of deep anesthesia. Cervical dislocation was performed only after the animals completely lost consciousness and pain response to ensure no pain or distress during the procedure, followed by dissection to collect colon tissues and intestinal contents. Colonic tissues were gently rinsed with approximately 5 mL of PBS to remove residual luminal contents and then fixed in 4% paraformaldehyde for subsequent histopathological and immunofluorescence analyses. Intestinal contents were immediately stored at − 80 °C after collection for 16S rRNA gene sequencing analysis of the gut microbiota.

All operators involved in animal experiments had received standardized training in laboratory animal husbandry and operation, and possessed corresponding professional competence and qualification for animal experimentation. All animal experimental procedures, including euthanasia methods, were in compliance with the relevant provisions of the Measures for Ethical Review of Science and Technology (Guo Ke Fa Jian [2023] No. 167) and were reviewed and approved by the Ethics Committee of the Laboratory Animal Center of Zhengzhou University (Approval No.: ZZU-LAC20241217[01]).

### Therapeutic assessment in DSS-Induced colitis model

The colon samples collected from dissection on day 14 (as described in Sect. “Preparation and optimization of sustained-release granules”) were washed with PBS and fixed in 4% paraformaldehyde for hematoxylin and eosin (HE) staining to evaluate histopathological changes. The intestinal contents were stored at −80 °C for subsequent microbiota analysis.

### Immunofluorescence analysis of inflammation resolution

To evaluate anti-inflammatory effects of the sustained-release granules, immunofluorescence staining was performed. Tissue sections were incubated overnight at 4 °C with primary antibodies targeting CD86 (a marker of pro-inflammatory M1 macrophages), CD206 (a marker of anti-inflammatory M2 macrophages), and F4/80 (a pan-macrophage marker in mice), followed by 1-hour incubation at 37 °C with appropriate secondary antibodies. Fluorescence microscopy was used for imaging and analysis of macrophage polarization.

### 16S rRNA gene sequencing analysis

Gut microbiota diversity was analyzed using 16S rRNA gene sequencing. At the end of the experimental period, fresh intestinal content samples were collected from mice, immediately frozen in liquid nitrogen, and stored at −80 °C until analysis. Microbial genomic DNA was extracted from the samples, and the V3–V4 hypervariable regions of the bacterial 16S rRNA gene were amplified using universal primers. The resulting amplicons were sequenced on an Illumina platform. Raw sequencing reads were subjected to quality filtering, denoising, and merging, and operational taxonomic units (OTUs) were clustered at 97% sequence similarity. Taxonomic assignment was performed using a reference database. Alpha diversity indices, including the Shannon and Chao1 indices, were calculated to evaluate microbial richness and diversity. Beta diversity was assessed using principal coordinate analysis (PCoA) based on Bray–Curtis distances to compare microbial community structures among different groups. The raw date were uploaded to the NCBI SRA database (Accession Number: PRJNA1416471).

### Statistical analysis

Body weight and colon length were measured individually for each mouse. Histological scores were evaluated in a blinded manner based on standard criteria. All experiments were repeated in triplicate and all experimental data were expressed as mean ± standard deviation (SD). GraphPad Prism software and Oringin software were used for statistical analysis. Comparisons between two groups were analyzed using Student’s t-test. For multiple group comparisons, one-way analysis of variance (ANOVA) followed by Tukey’s multiple comparison test was applied. Differences were considered statistically significant at *p* < 0.05.

## Results

### Disintegration test in simulated gastric and intestinal fluids

Inhibition zone assays revealed that all tested coating materials exhibited negligible antibacterial activity against *L. plantarum*, indicating their suitability as probiotic carriers. Particles were prepared from these materials for subsequent solubility analysis.In vitro disintegration and dissolution are two distinct but sequential processes for solid oral dosage forms. In the present study, the disintegration behavior of the sustained-release granules was first evaluated, followed by the assessment of bacterial release after disintegration in simulated gastrointestinal environments.

**Fig. 1:**
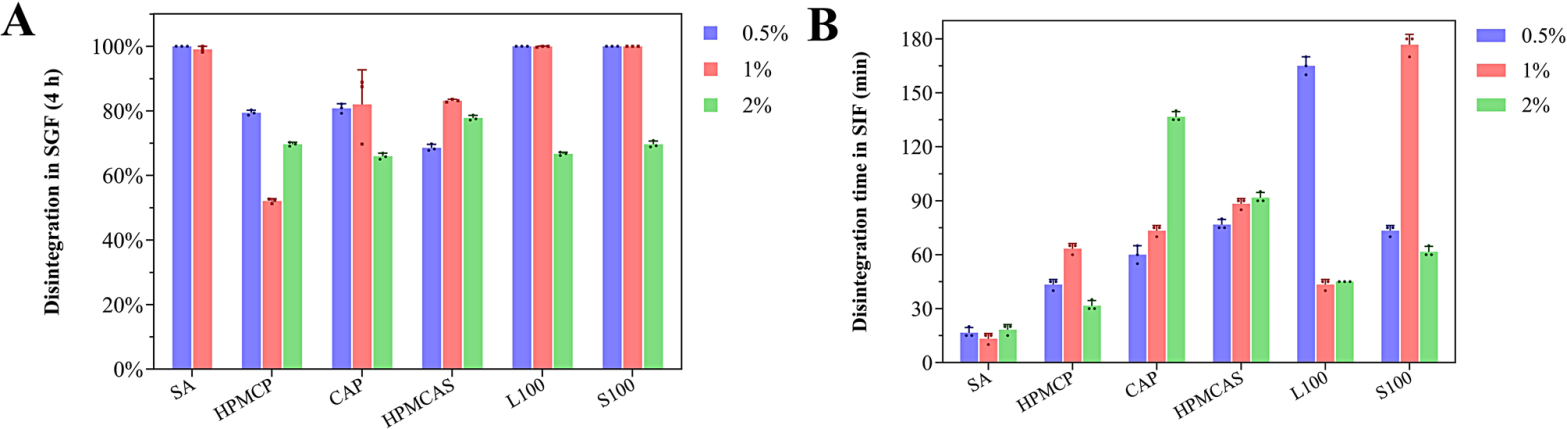
In vitro disintegration behavior of *Lactiplantibacillus plantarum* sustained-disintegration granules. (**A**) cumulative disintegration of different granule formulations in SGF (*n* = 3); (**B**) time required for complete disintegration of granules in SIF (*n* = 3)

Both HPMCP and L100 granules exhibited delayed disintegration in simulated gastric fluid (SGF). At concentrations of 1 and 2%, HPMCP and L100 exhibited significantly lower disintegration rates compared to CAP, HPMCAS, and S100, and performed better than SA, indicating superior protective effects in simulated gastric fluid and effective delay in premature disintegration. These findings demonstrate the favorable enteric properties of HPMCP and L100, which contribute to minimizing probiotic loss during gastric transit. Additionally, the disintegration times of HPMCP and L100 at 1 and 2% concentrations ranged from 60 to 100 minutes in simulated intestinal fluid—moderate compared to S100, which disintegration too rapidly, and CAP, which exhibited prolonged disintegration times nearing 180 minutes at 2%. Interestingly, granules containing 0.5% HPMCP also exhibited good acid resistance, and 0.5% L100 granules performed comparably to the 1% formulation under acidic conditions. However, the 0.5% HPMCP granules disintegrated too rapidly in SIF, failing to achieve the desired slow intestinal release, whereas 0.5% L100 granules disintegrated too slowly in SIF, potentially limiting their efficacy in the intestine. Considering these limitations, both formulations were excluded from further investigation. This suggests that HPMCP and L100 provide a well-balanced disintegration profile in the intestinal environment, achieving sustained but timely probiotic disintegration without excessive delay. In contrast, under simulated intestinal fluid (SIF) conditions, the disintegration times were significantly shortened, and the disintegration amounts reached complete disintegration thresholds. By comparing the cumulative disintegration in SGF over 4 hours (Fig. 1A) and the time required for complete disintegration in SIF (Fig. 1B), it is evident that *L. plantarum* sustained-disintegration granules formulated with 1% HPMCP and 1% L100 successfully prevented premature disintegration in the gastric phase while enabling rapid disintegration and full disintegration in the intestinal phase.

This dual-phase disintegration behavior provides a crucial advantage for enhancing probiotic survival through the gastrointestinal tract, thereby significantly improving the intestinal delivery efficiency and in vivo efficacy of the sustained-release probiotic granules. Based on these findings, HPMCP and Eudragit L100 were identified as enteric coating materials for sustained-disintegration probiotic delivery systems.

### Determination of bacterial release after simulated intestinal fluid treatment

The total number of viable bacteria in the granules was determined before and after treatment with simulated gastric and intestinal fluids, and the release rate was calculated.

**Fig. 2:**
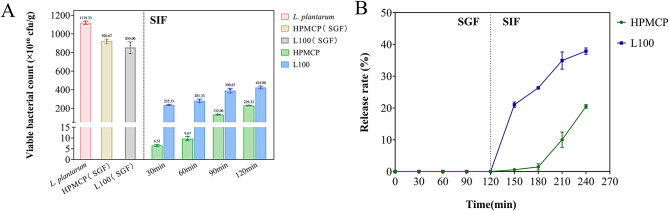
(**A**) initial viable counts of *Lactiplantibacillus plantarum* powder, survival of HPMCP–*L. plantarum* and L100–*L. plantarum* after 2 h in SGF (*n* = 3), and time-dependent viable counts of HPMCP–*L. plantarum*, and L100–*L. plantarum* in SIF (*n* = 3). (**B**) percentage of viable bacteria released from HPMCP–*L. plantarum* and L100–*L. plantarum* in SGF and SIF at different time points (*n* = 3)

Figure 2 illustrates the release behavior of the granules in simulated intestinal fluid following 2 hours of treatment in simulated gastric fluid. As shown in Fig. 2A, over 70% of *Lactiplantibacillus plantarum* remained viable after 2 hours of exposure to the simulated gastric environment, indicating that the encapsulated probiotics were effectively protected under acidic conditions. As shown in Fig. 2B, the prepared sustained-release *L. plantarum* granules exhibited excellent controlled-release properties, with release persisting for more than 2 hours. In addition, the initial release rate of *L. plantarum* from L100-based granules was faster than that from HPMCP-based granules. This feature is consistent with the more rapid disintegration observed for L100 granules in the disintegration tests, suggesting that they may exert their effects earlier in the intestine, which could be beneficial for timely treatment of colitis. Meanwhile, HPMCP-based granules demonstrated prolonged release and superior controlled-release performance, allowing for extended bacterial delivery and potentially providing more sustained therapeutic effects for colitis with reduced dosing frequency and improved convenience. Furthermore, considering that gastric acidity itself can affect microbial viability, subsequent histopathological analyses further confirmed that the sustained-release granules may offer enhanced therapeutic outcomes in colitis.

### Morphological characterization of granules

**Fig. 3:**
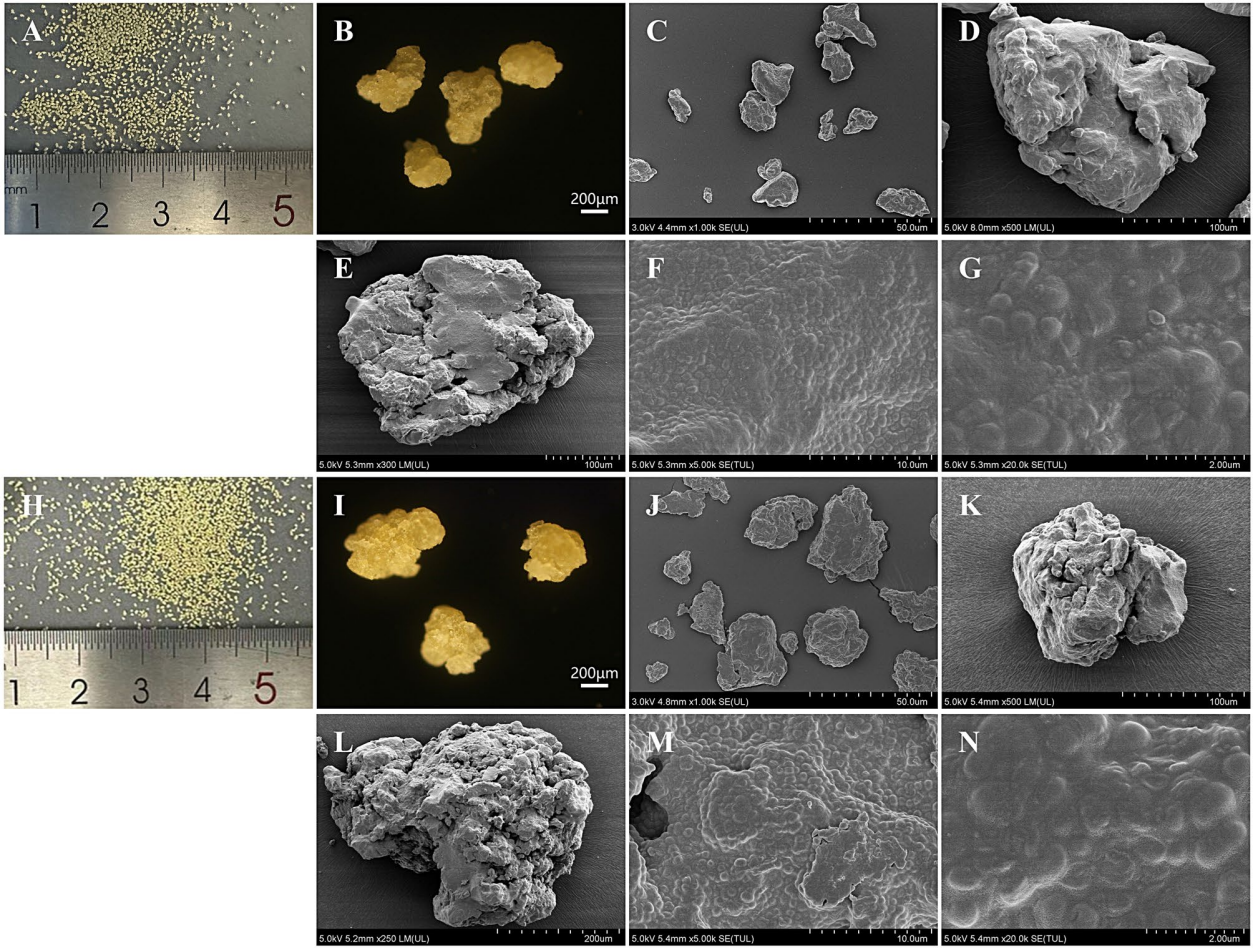
Scanning electron microscopy (SEM) analysis of the sustained-release granules. (**A-B**) Macroscopic image of HPMCP–*Lactiplantibacillus plantarum* granules and the corresponding magnified image at 100×. (**C–D**) SEM micrographs of HPMCP–*L. plantarum* granules at different magnifications. (**E–G**) SEM micrographs of the internal structure of physically fractured HPMCP–*L. plantarum* granules at different magnifications. (**H-I**) Macroscopic image of HPMCP–*Lactiplantibacillus plantarum* granules and the corresponding magnified image at 100× (**J–K**) SEM micrographs of L100–*L. plantarum* granules at different magnifications. (L–N) SEM micrographs of the internal structure of physically fractured L100–*L. plantarum* granules at different magnifications

The HPMCP–*Lactiplantibacillus plantarum* sustained-release granules (Fig. 3A and B) and L100–*L. plantarum* sustained-release granules (Fig. 3H and I) exhibited uniform geometric shapes at the macroscopic level. The particle size distribution of both sustained-release granules was narrow and uniform, ranging from 40 to 80 mesh (180–425 μm). Notably, over 85% of the granules were concentrated within the 40–60 mesh range (250–425 μm). SEM observation indicated that the surfaces of HPMCP–*L. plantarum* granules (Fig. 3C and D) and L100–*L. plantarum* granules (Fig. 3J and K) were smooth, intact, and uniform in texture. After physical grinding and fracture, the internal cross-sections of both types of granules displayed relatively rough and porous structures (Fig. 3E and L). At higher magnifications (5000× and 20,000×), the granules were found to consist of numerous fine spherical subunits, in which HPMCP or L100 served as the sustained-release matrix material, encapsulating uniformly distributed sucrose as a filler along with *L. plantarum* cells. These subunits were tightly connected, forming a multi-spherical sustained-release structure. Even when the outer layers were partially damaged, this architecture effectively protected the majority of bacterial cells’ physiological activity, ensuring that viable bacteria were primarily released upon reaching the intestinal environment.

### Therapeutic efficacy of granules

#### Resolution of inflammation

**Fig. 4:**
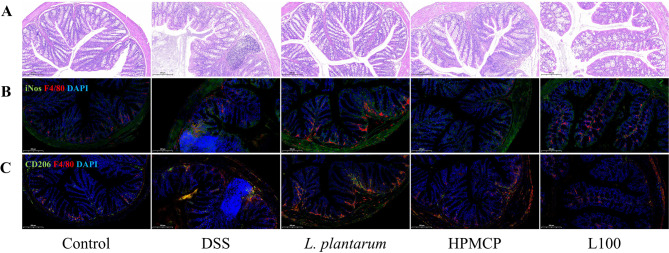
Immunofluorescence staining results of different treatment groups in mice after therapy completion. (**A**) HE staining of colon sections across treatment groups post-treatment; (**B**) Representative immunofluorescence images of M1^+^ cells (green, iNOS^+^) and F4/80^+^ macrophages (red) in colon tissue; (**C**) Representative immunofluorescence images of M2^+^ cells (green, CD206^+^) and F4/80^+^ macrophages (red) in colon tissue

Dextran sulfate sodium salt (DSS) administration induces clinical manifestations in mice that closely resemble ulcerative colitis (UC), including body weight loss, diarrhea, and hematochezia [18, 19]. In this study, a DSS-induced colitis model was employed to evaluate the therapeutic efficacy of the orally administered sustained-release pellet formulation. Compared to the normal group, DSS-treated mice showed significant weight loss and histological signs of colitis, including epithelial damage, crypt loss, and inflammatory infiltration, confirming successful model induction (Fig. S1). As shown in Fig. 4A, the HPMC and L100 granule treated groups showed different degrees of epithelial structure restoration compared to the traditional administration method of *Lactiplantibacillus plantarum* powder by gavage with solution. In particular, administration of HPMCP sustained-release granules significantly reduced the level of inflammatory cell infiltration and revealed histological structures nearly indistinguishable from those of the normal control group. Compared with the DSS group and the bacterial powder group, the granule-treated groups exhibited reduced inflammatory cell infiltration, mucosal damage, and crypt destruction in colonic tissues, as evidenced by hematoxylin and eosin (H&E) staining and histological scoring (Fig. S1H). As illustrated in Fig. 4B and 4C, standard markers including iNOS^+^, CD206, and F4/80 were used to identify M1 and M2 macrophages. DSS-induced colitis mice exhibited increased macrophage infiltration in the colon, with both M1 and M2 macrophage populations elevated compared to the control group. Oral administration of HPMCP and L100 sustained-release granules led to a reduction in M1 macrophages and a concomitant increase in M2 macrophages. These findings indicate that both HPMCP and L100 granules modulate the M1/M2 macrophage polarization balance, particularly promoting M2 polarization (Fig. S2). This suggests that enhancing M2 polarization through *L. plantarum* sustained-release granules may represent a potential therapeutic strategy for inflammatory bowel disease (IBD).

#### Gut microbiota diversity and composition

A growing body of evidence indicates that the gut microbiota plays a direct role in the pathogenesis of inflammatory bowel disease (IBD) [20, 21]. Distinct microbial communities within the colon form an interactive and balanced ecosystem, which becomes disrupted in the context of IBD and other colonic disorders [22, 23]. Therefore, this study investigated whether *Lactiplantibacillus plantarum* sustained-release pellets could restore gut microbial homeostasis in a murine model.

**Fig. 5:**
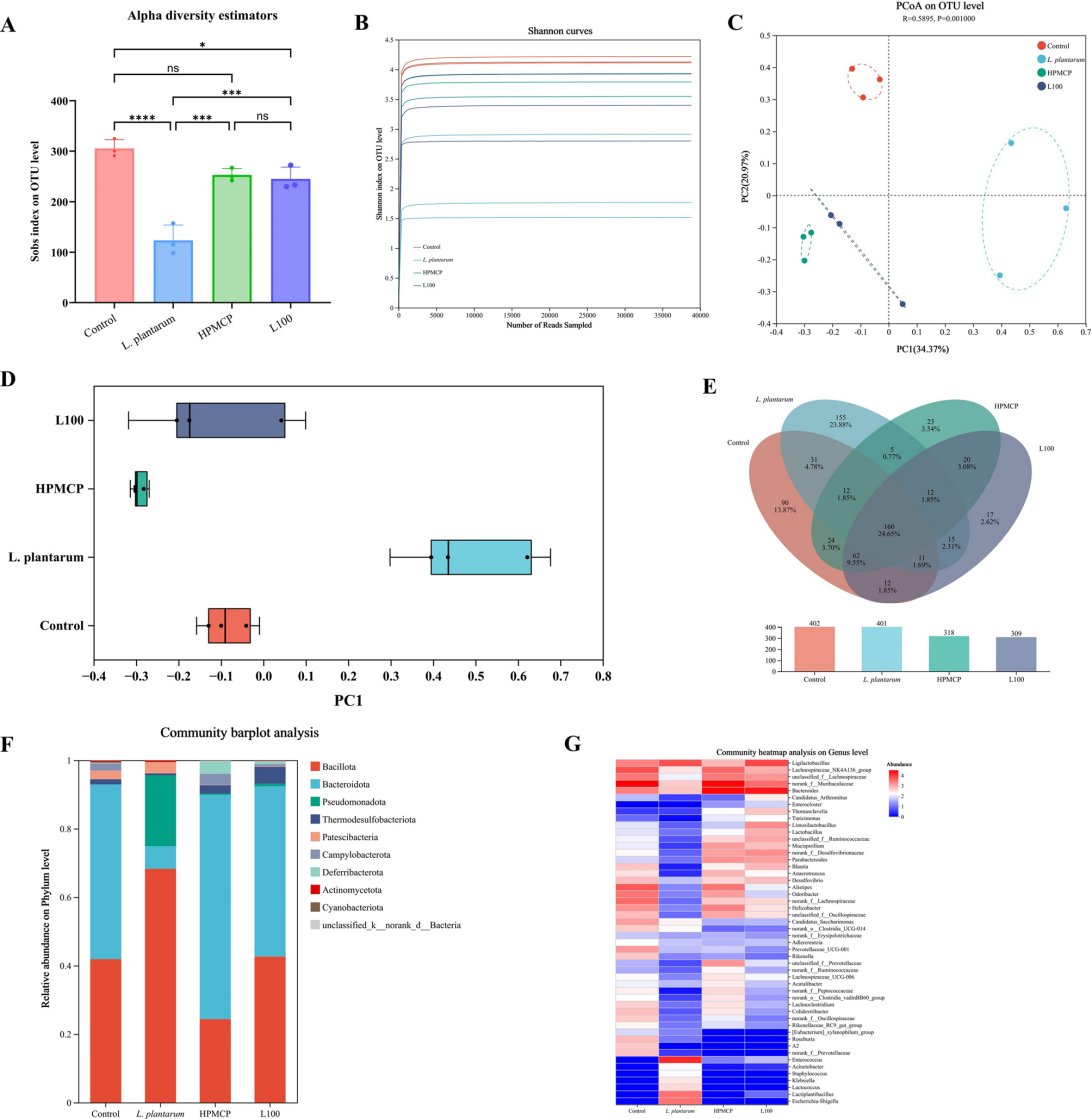
16S rRNA gene sequencing of gut microbiota in different treatment groups of mice (*n* = 3). (**A**) observed species index (Sobs) at the operational taxonomic unit (OTU) level across treatments; (**B**) α-diversity analysis displayed by Shannon curves at OTU level; (**C**) Principal coordinate analysis (PCoA); (**D**) box plots of PCoA distances between treatment groups; (**E**) venn diagram of common and unique bacterial species among groups; (**F**) microbial composition at phylum level; (**G**) heatmap of relative abundance of microbiome at genus level. Data are presented as mean ± SD. Statistical significance was analyzed using one-way ANOVA followed by Tukey’s multiple comparisons test. (*n* = 3; *p < 0.05, **p < 0.01, ***p < 0.001, ****p < 0.0001)

Alpha diversity is an ecological metric used to assess the richness and evenness of taxa within individual samples [24]. Greater microbial species richness in an ecosystem is generally reflected by higher alpha diversity indices. As shown by the Sobs index results (Fig. 5A), alpha diversity was significantly reduced in the probiotic powder group compared to the normal control group. This decline may be attributed to DSS-induced inflammation, which promotes the overgrowth of harmful intestinal bacteria while suppressing the proliferation of commensal microbes, ultimately leading to reduced microbial abundance. In contrast, oral administration of HPMCP- and L100-based sustained-release pellets markedly increased Sobs index values, with statistically significant differences compared to the DSS group. Notably, these results suggest that treatment with the sustained-release formulations effectively restored microbial species diversity toward levels observed in healthy controls.

Principal coordinate analysis (PCoA) showed significant differences in similarity between the control group and the DSS-induced model group. PCoA (Fig. 5C and 5D) revealed distinct clustering of treatment groups. Microbiota profiles in the HPMCP and L100 groups were more similar to the normal group than to the DSS or powder groups, indicating restorative effects.

To elucidate the compositional structure of the gut microbiota, microbial differences were analyzed at both the phylum and genus levels [25]. At the phylum level (Fig. 5F), six dominant bacterial phyla were identified, among which Firmicutes and Bacteroidetes represented the two most abundant taxa, collectively accounting for over 90% of the total microbiota and dominating across all samples. Compared to the normal control group, the probiotic powder group exhibited an increased relative abundance of Firmicutes and a decreased abundance of Bacteroidetes. The Firmicutes-to-Bacteroidetes (F/B) ratio is widely recognized as a key indicator of microbial dysbiosis in the gut. In contrast, treatment with HPMCP- and L100-based sustained-release pellets resulted in a decreased abundance of Firmicutes and an increased abundance of Bacteroidetes relative to the powder group, indicating a regulatory effect on gut microbiota composition. Notably, the L100 group demonstrated the most pronounced modulation of the Firmicutes and Bacteroidetes populations in colitis-induced mice, with microbial profiles approaching those observed in the healthy control group.

At the genus level, microbial community composition was further characterized using a heatmap analysis (Fig. 5G). In the normal group, genera such as Bacteroides, Alistipes, and Odoribacter were present at relatively high abundances. In contrast, the probiotic powder group exhibited a marked increase in potentially pathogenic genera including Escherichia-Shigella, Enterococcus, and Staphylococcus, consistent with findings shown in Fig. 5C, suggesting a detrimental impact on intestinal health. In the HPMCP-treated group, beneficial genera such as Candidatus_Arthromitus and Turicibacter emerged as dominant taxa with increased abundance. In the L100 group, there was a significant enrichment of Roseburia, a known short-chain fatty acid-producing genus, along with A2 and unclassified taxa within the Prevotellaceae family. These observations indicate that treatment with HPMCP- and L100-based sustained-release pellets modulated the gut microbiota by selectively enriching beneficial bacterial populations, thereby contributing to improved microbial community function. Collectively, these findings suggest that the oral administration of *L. plantarum* sustained-release pellets promotes microbial homeostasis and supports therapeutic efficacy in the context of inflammatory bowel disease.

## Discussion

In this study, a novel sustained-release and enteric-coated delivery system for *Lactiplantibacillus plantarum* was successfully developed based on a cross-linked porous matrix constructed using pH-responsive polymers (HPMCP and Eudragit L100). This system incorporates a dual-protection mechanism of acid-resistant enteric coating and controlled-release internal matrix, which significantly improves the survival rate and therapeutic efficacy of the probiotic in a dextran sulfate sodium (DSS)-induced acute colitis mouse model, and demonstrates superior performance compared to traditional oral probiotic powders.

Clinically, oral administration of *Lactiplantibacillus plantarum* powder has been demonstrated to exert beneficial effects on ameliorating colitis symptoms [2]. However, its efficacy is constrained by significant degradation in gastric acid and bile salts, leading to reduced viable bacteria reaching the colon and suboptimal colonization [26, 27]. Consequently, achieving the desired therapeutic outcomes often necessitates prolonged, frequent, and high-dose administration regimens (e.g., multiple times daily) [28]. This not only increases the patient compliance burden and treatment costs but also carries the potential for discomfort due to excessive intake. In contrast, the enteric-coated sustained-release granule strategy proposed in this study utilizes the “smart” protection afforded by HPMCP (hydroxypropyl methylcellulose phthalate) and L100 enteric coatings. In addition to HPMCP and L100, several other polymeric materials, including SA, CAP, HPMCAS, and S100, were evaluated to compare their behavior under simulated gastric and intestinal conditions. Formulations based on SA exhibited rapid disintegration in SGF due to their hydrophilicity and limited acid resistance, resulting in premature probiotic release. In contrast, cellulose-derived enteric polymers such as HPMCP and CAP provided improved acid protection; however, their disintegration behavior in SIF was less consistent, likely due to pH sensitivity and variations in film integrity. HPMCAS showed moderate resistance in SGF and gradual disintegration in SIF, reflecting its amphiphilic properties. Among the polymers tested, L100 and S100 demonstrated superior pH-responsive behavior, maintaining relative stability under acidic conditions while effectively disintegrating in SIF. Notably, HPMCP and L100 achieved a more balanced performance, offering sufficient gastric protection without excessively delaying intestinal release, supporting their selection as the optimal coating materials in this study. This effectively shields the probiotics from gastric acid erosion, as confirmed by minimal release in simulated gastric fluid during in vitro dissolution testing, thereby ensuring the safe passage of the majority of viable bacteria through the stomach. Upon entry into the near-neutral or weakly alkaline intestinal environment (pH > 5.5–6.0), the coating rapidly dissolves. Scanning electron microscopy (SEM) analysis further revealed that the granules possess an internal sucrose-filled cross-linked porous matrix, facilitating sustained release rather than burst release. This combined mechanism of gastric protection and colon targeting plays a crucial role in ensuring the overall therapeutic efficacy of the formulation.

Compared with conventional probiotic powders, the proposed delivery system combines a unique physical architecture (cross-linked porous matrix) with a chemical barrier (pH-responsive polymer coating), thereby establishing an efficient platform for dual protection and controlled release. Notably, under equivalent probiotic dosing, the HPMCP- and L100-based sustained-release granules developed in this study demonstrated significantly superior therapeutic efficacy against DSS-induced acute colitis compared to direct oral administration of freeze-dried *Lactiplantibacillus plantarum* powder. This therapeutic advantage is not solely attributed to the probiotic itself, but more importantly to the optimized gastrointestinal delivery and targeted release strategy afforded by the granule system. Such a strategy effectively reduces dosing frequency and total dosage, enhances patient compliance, and achieves equal or even improved therapeutic outcomes. Therefore, this study confirms from multiple aspects that the delivery system is more effective and demonstrates superior performance.

Histological evaluation (Fig. 4A) indicated that both the HPMCP- and L100-treated granule groups exhibited partial improvement in epithelial organization compared with the DSS group. Notably, treatment with HPMCP-based sustained-release granules was associated with a marked attenuation of inflammatory cell infiltration, and the overall colonic architecture appeared largely preserved when compared with the DSS model group. Relative to both the DSS group and the conventional probiotic powder group, mice receiving sustained-release granules exhibited reduced inflammatory infiltration, less severe mucosal injury, and improved preservation of crypt structures, with the HPMCP formulation showing the most pronounced histological improvement. H&E staining suggested that, compared with free probiotic powder, both HPMCP- and L100-based sustained-release granules more effectively maintained epithelial integrity and mitigated tissue damage in DSS-induced colitis mice. These histopathological findings indicate a potential advantage of sustained-release formulations in alleviating colonic inflammation. Consistently, immunofluorescence analysis provided complementary evidence supporting the observed reduction in inflammatory responses.

Immunofluorescence analysis (Fig. 4B, C) indicated a reduced presence of pro-inflammatory M1 macrophage markers (iNOS^+^/F4/80^+^) and an increased presence of M2-associated markers (CD206^+^/F4/80^+^) in granule-treated groups compared with the DSS group, whereas these changes were less pronounced in the bacterial powder group. These findings suggest that sustained-release granules may enhance the anti-inflammatory effects of probiotics, potentially through improved delivery of viable bacteria and prolonged residence in the intestinal tract [26, 29]. In addition to the histopathological and immunofluorescence evidence indicating attenuation of colonic inflammation, modulation of the intestinal microbiota represents another important indicator for evaluating therapeutic improvement in colitis.

We conducted high-throughput 16S rRNA gene sequencing to investigate changes in the intestinal microbiota. 16S rRNA gene sequencing highlighted another key advantage: while powder minimally improved DSS-induced reductions in alpha diversity analysis (Fig. 5A, B) indicated that both granule-treated groups showed an increasing trend in microbial richness and diversity compared with the DSS group. PCoA analysis (Fig. 5C, D) revealed a partial shift of the microbial community structure in granule-treated groups toward that of the normal control. Granules also more effectively normalized the dysbiotic Bacillota/Bacteroidetes ratio elevated by powder (Fig. 5F). At the genus level (Fig. 5G), granule groups uniquely enriched beneficial taxa (e.g., Candidatus_Arthromitus, Turicibacter with HPMCP; Roseburia, Prevotellaceae with L100), linked to anti-inflammation and barrier function [30, 31], contrasting with higher potential pathogens (Escherichia-Shigella, Enterococcus) in powder recipients. Collectively, the sustained-release strategy, by ensuring viable probiotic delivery and persistence, profoundly reshapes the gut microbiota towards a healthier, anti-inflammatory state, underpinning its enhanced efficacy.

Crucially, at the same dosage, our delivery platform outperformed traditional powder formulations in almost all indicators, including histopathology, immune regulation, and microbiota normalization, indicating that the delivery technology enhances probiotic efficacy more effectively than simply increasing the dose. Interestingly, the therapeutic effect of this delivery system may also have considerable advantages compared to current chemical treatments for colitis.

Current pharmacological treatments for intestinal inflammation, particularly inflammatory bowel disease (IBD), primarily include 5-aminosalicylic acid (5-ASA) derivatives, corticosteroids, immunosuppressants (e.g., azathioprine, methotrexate), and biologics such as anti-TNF-α monoclonal antibodies [32]. Although these agents can be effective, they are often associated with serious adverse effects, including increased risks of infection, metabolic disturbances, bone marrow suppression, and long-term malignancy [33, 34]. In contrast, probiotics are live microorganisms that are generally regarded as safe, with side effects such as bloating or mild gastrointestinal discomfort usually being temporary and self-limiting. Beyond direct anti-inflammatory activity, the sustained-release granule system presented in this study offers a multi-targeted therapeutic approach. It not only delivers viable *Lactiplantibacillus plantarum* to the intestine but also facilitates the restoration of microbial homeostasis, the reinforcement of mucosal barrier integrity, and the modulation of the immune response, specifically by enhancing M2 macrophage polarization [35, 36]. Such mechanisms address the underlying pathophysiology of IBD rather than merely suppressing inflammation. For instance, 5-ASA acts mainly as a local anti-inflammatory agent and exhibits limited ability to regulate microbial dysbiosis. In this context, the superior epithelial repair observed in the granule-treated groups (as shown in Fig. 4A) underscores the microbiota-mediated benefits of *L. plantarum* and its metabolites, such as short-chain fatty acids, in promoting epithelial regeneration and mucosal healing [37]. These findings highlight the therapeutic potential of probiotic-based sustained-release systems as safer, more holistic alternatives or adjuncts to conventional pharmacological interventions.

Probiotic immunomodulation and microbiota restoration require time, potentially limiting their speed in controlling acute, severe inflammation compared to potent anti-inflammatories like corticosteroids or biologics. Consequently, probiotics alone are often insufficient for inducing remission in severe active IBD, which typically requires conventional agents [38]. Probiotic efficacy may also exhibit greater individual variation due to factors like baseline microbiota or genetics [39]. Nevertheless, in our DSS-induced acute colitis model (mimicking UC), the sustained-release granule system (particularly HPMCP granules) achieved near-normal levels of histological repair and microbiota restoration, alongside significant anti-inflammatory effects (e.g., macrophage polarization). The degree of histological repair and anti-inflammatory effects observed with the sustained-release granules, particularly HPMCP granules, in this acute DSS-colitis model appear comparable to the reported efficacy of first-line therapies like 5-ASA in managing mild-to-moderate ulcerative colitis [40], based on established clinical and preclinical knowledge. While 5-ASA remains a cornerstone for inducing and maintaining remission in this patient group, our granules demonstrated comparable efficacy in the model, coupled with a superior safety profile and the added benefit of microbiota modulation.

The superior therapeutic performance of our system stems from its innovative scaffold‑based architecture, which affords excellent protection and precise release of probiotics. In dissolution data (Fig. 2) confirm that HPMCP and L100 coatings confer excellent acid resistance, minimizing probiotic loss in simulated gastric fluid, while the cross‑linked porous matrix (SEM, Fig. 3) enables controlled, sustained release in the intestinal milieu [41] Unlike traditional enteric-coated tablets or capsules where failure of a single unit results in exposure of the entire payload to gastric acid, our multi-unit granules compartmentalize the risk so that damage to individual particles causes only minor probiotic loss while the intact granules continue to deliver viable cells [42]. Moreover, embedding probiotics and sucrose fillers uniformly within a micro‑/nano‑scale polymeric network creates local protective microenvironments and controlled‑release pathways that simple powder blending cannot achieve. Compared with microencapsulation techniques, which often require high temperature, organic solvents, or shear stress that jeopardize cell viability, our Wet granulation and low‑temperature extrusion processes employ well‑established pharmaceutical excipients (HPMCP, L100, sucrose) and are readily scalable under GMP conditions. These features collectively position the scaffold‑based granule system as a robust, manufacturable platform for advanced probiotic delivery.

Various probiotic strains including Bifidobacterium species and other *Lactobacillus* species such as *Lacticaseibacillus rhamnosus* and *Lactobacillus acidophilus*, as well as multi-strain formulations and synbiotics which are combinations of probiotics and prebiotics, have been widely investigated for the treatment of intestinal inflammation [43–45]. Certain combinations have demonstrated potential synergistic effects [46]. While *L. plantarum*, the strain selected in this study, possesses intrinsic acid and bile resistance, adhesive capacity, and immunomodulatory properties [47], the principal contribution of this work lies not in contesting the efficacy of other strains or combinations, but in presenting a broadly applicable and efficient delivery platform. The enteric sustained-release granule system developed herein, based on a cross-linked skeleton structure and low-temperature extrusion-coating process, offers excellent compatibility and is theoretically adaptable for the delivery of other individual strains, multi-strain consortia, or even synbiotic preparations [48]. Future studies could explore optimized probiotic combinations such as *Lactiplantibacillus plantarum* co-administered with selected Bifidobacterium species using this platform to further enhance therapeutic outcomes and potentially surpass the efficacy of some existing commercial formulations. Crucially, the system ensures both the stability of each strain during processing and their coordinated release within the gastrointestinal tract.

Maintaining a high activity of probiotics during processing cannot be ignored either. Our granule fabrication employed meticulously maintained low temperatures (35–40 °C during wet granulation and drying), minimizing heat-induced inactivation. This contrasts sharply with processes like spray drying microencapsulation, where high inlet temperatures (150–200°C) often cause significant viability loss despite lower outlet temperatures [49, 50]. Furthermore, we proactively addressed potential excipient toxicity: all candidate coating materials (SA, HPMCP, CAP, HPMCAS, L100, S100) were pre-screened for antimicrobial activity against *L. plantarum* using an agar diffusion assay. No significant inhibition was observed at relevant concentrations, confirming the biocompatibility of the selected polymers (HPMCP, L100). Solvents (methanol-acetone for HPMCP, 75% ethanol for L100/S100) were also chosen for minimal antimicrobial impact. This crucial step, often overlooked when selecting materials based solely on physicochemical properties [51], ensures excipients do not harm probiotics during processing or storage. Collectively，this low-temperature processing and biocompatible excipient/solvent selection strategy effectively maintained activity during the production process, resolving the issue of a significant decline in live bacteria counts reported in other studies involving probiotic microcapsules or particles after preparation [49, 52], and providing a basis for the formulation’s efficacy.

The enteric-coated sustained-release granule system developed herein represents a promising next-generation platform for oral probiotic delivery. Its gastric protection and colon-targeted sustained release are particularly well-suited for the maintenance therapy of chronic inflammatory bowel diseases (IBD, including UC and CD) [53], potentially reducing relapse frequency/severity and dependence on conventional drugs by ensuring continuous delivery of viable probiotics to the inflamed site. The inherent compatibility and scalability of the granule matrix and coating process readily allow adaptation for delivering other single probiotic strains or designing advanced formulations. This includes multi-strain probiotic consortia leveraging synergistic interactions (e.g., *L. plantarum* + *Bifidobacterium spp.*) [44, 46] or synbiotics by incorporating prebiotics (e.g., FOS, inulin) into the matrix to selectively enhance probiotic activity in situ [54]. Taken together, this modular, scalable platform offers a versatile foundation for the development of advanced probiotic.

### Limitations of the work

This study has achieved preliminary progress in the development and validation of an enteric-coated sustained-release delivery system for *Lactiplantibacillus plantarum*, there remains considerable room for optimization and further expansion. Animal experiments were strictly performed in accordance with the double-blind principle and the 3 R principles (replacement, reduction, refinement), with the minimum sample size adopted. Despite the relatively small sample size, the results still showed favorable trends; future studies could increase the sample size to strengthen the persuasiveness of the conclusions. The sustained-release micro-particles constructed in this study exhibited favorable alleviating effects on DSS-induced acute colitis models; however, validation in colitis models induced by other etiological factors is still lacking. Further verification is warranted in future research to comprehensively evaluate the therapeutic efficacy of the delivery system. Although there are discrepancies between simulated systems and the real physiological environment, the overall results still provide valuable implications. Subsequent studies may combine refined in vitro–in vivo correlation investigations to improve the accuracy of the evaluation system. Only a single strain of *Lactiplantibacillus plantarum* was selected in this study, and the applicability of this delivery system to other probiotic strains has not been explored; thus, its application scope needs to be further expanded. Research on sustained-release particles loaded with multiple probiotic strains is currently underway. Animal experiments were conducted in a standardized laboratory environment, with irrelevant variables strictly controlled. Nevertheless, the human clinical setting is highly complex, and factors such as individual physiological differences, lifestyle habits, and concurrent medications may affect the in vivo performance of the formulation, leading to limited clinical extrapolation of the findings.

To address the above limitations, future studies should expand the sample size, optimize the experimental design, perform validation in chronic colitis models, and conduct repeated experiments with multiple strains, so as to improve the generalizability of the conclusions and their value for clinical translation.

## Conclusions

In this study, we successfully developed and validated an enteric-coated, sustained-release granule system for *Lactiplantibacillus plantarum*, based on an innovative cross-linked porous matrix formed by HPMCP and L100. The system employs a dual protective mechanism: a gastric pH-responsive coating shields the probiotics from acid-induced damage, while the intestinal matrix enables controlled, sustained release. This approach represents a significant advancement in oral probiotic delivery, markedly enhancing survival through the harsh gastrointestinal environment and ensuring targeted, prolonged release in the colon. In a DSS-induced murine colitis model, at equivalent viable cell doses, the granules exhibited substantially superior therapeutic efficacy compared with conventional freeze-dried powder, including enhanced histological repair, effective immunomodulation (e.g., promotion of M2 macrophage polarization), and comprehensive restoration of gut microbiota homeostasis. These benefits are closely associated with the improved gastrointestinal survival and efficient intestinal delivery of viable probiotics afforded by the formulation. Importantly, high bacterial viability was maintained during manufacturing through carefully controlled low-temperature processing and the use of rigorously screened, biocompatible, non-antimicrobial excipients.

The matrix-based multi-particulate system offers distinct advantages over existing technologies (e.g., single-unit enteric-coated tablets/capsules, microcapsules), including superior protection uniformity, resilience against localized failure, gentle processing, and enhanced manufacturability.

Beyond enabling efficient *L. plantarum* delivery, this versatile platform technology may also have potential for delivering other single probiotics, multi-strain consortia, or synbiotics. Its possible applications could include maintenance therapy for IBD, prevention of antibiotic-associated diarrhea (AAD), management of *C. difficile* infection (CDI), and other dysbiosis-related conditions. Further work on long-term stability, large-scale manufacturing, and well-designed clinical trials would be needed to explore the feasibility of this next-generation delivery system. Such efforts may ultimately contribute to advances in gastrointestinal health management and personalized nutritional interventions, though additional validation is required

## Electronic supplementary material

Below is the link to the electronic supplementary material.

Supplementary material 1

## Data Availability

The sequencing data generated in this study have been deposited in the NCBI Sequence Read Archive (SRA) database under the accession number (PRJNA1416471). All other data supporting this research can be found in the article or its supplementary materials.

## Abbreviation

5-ASA: 5-aminosalicylic acid
AAD: Antibiotic-associated diarrhea
CAP: Cellulose acetate phthalate
CD86: Cluster of differentiation 86
CD206: Cluster of differentiation 206
CDI: *Clostridioides difficile* infection
DSS: Dextran sulfate sodium salt
F4/80: F4/80 antigen (EMR1, mouse macrophage marker)
HE: Hematoxylin and eosin
HPMCAS: Hydroxypropyl methylcellulose succinate
HPMCP: Hydroxypropyl methylcellulose phthalate
IBD: Inflammatory bowel disease
iNOS: Inducible nitric oxide synthase
L100: Eudragit L100
*L. plantarum*: *Lactiplantibacillus plantarum*
MRS: De Man, Rogosa and Sharpe (broth/agar)
OTU: Operational taxonomic unit
PBS: Phosphate-buffered saline
PCoA: Principal coordinate analysis
S100: Eudragit S100
SA: Sodium alginate
SEM: Scanning electron microscopy
SGF: Simulated gastric fluid
SIF: Simulated intestinal fluid
Sobs: Number of observed species (or OTUs)
UC: Ulcerative colitis
