# Supplementary material for: Sustained-release enteric formulations of Lactiplantibacillus plantarum based on granulation technology: preparation and therapeutic evaluation in acute colitis
Source: BMC Biotechnol. 2026 Apr 21;26:73. doi: 10.1186/s12896-026-01157-7 (PMC13231606; doi:10.1186/s12896-026-01157-7)
Supplement: Supplementary file 1 — Supplementary material 1 [file 12896_2026_1157_MOESM1_ESM.zip › 12896_2026_1157_MOESM1_ESM/Supplementary.docx]

**Supplementary**

**Table 1 DAI Scoring Criteria**

| Score | Percentage of Weight Loss | Stool Consistency | Stool Bleeding |
| --- | --- | --- | --- |
| 0 | 0 | Normal | No bleeding |
| 1 | 1-5% | Soft stool | Minimal bleeding |
| 2 | 5-10% | Mucus-like stool | Mild bleeding |
| 3 | 10-20% | Watery stool | Moderate bleeding |
| 4 | ＞20% |  | Gross visible bleeding |

**Table 2 Disease Activity Index (DAI) severity grading**

| Total Score Range | Disease Activity Level |
| --- | --- |
| 0-1 points | Completely normal (no disease activity) |
| 1-5 points | Mild disease activity |
| 5-9 points | Moderate disease activity |
| 9-12 points | Severe disease activity |

**Table 3 Histology Scoring**

| Score | Ulceration | Epithelial Cell Changes | Inflammatory Infiltration |
| --- | --- | --- | --- |
| 0 | No ulceration | Normal | None |
| 1 | Focal ulceration | Goblet cell loss | Pericryptal infiltration |
| 2 | Multiple ulcerations | Extensive goblet cell loss | Infiltration reaching the muscularis mucosae |
| 3 | Diffuse ulceration | Crypt loss | Diffuse infiltration of the muscularis mucosae, mucosal thickening |
| 4 | Diffuse ulceration with deep injury | Extensive crypt loss or polypoid regeneration | Infiltration reaching the submucosa |

**Table 4 Histological severity grading**

| Total Score Range | Severity Level | Description |
| --- | --- | --- |
| 0 | Normal | No significant pathological changes |
| 1-3 | Mild | Mild epithelial damage and/or mild inflammation |
| 4-7 | Moderate | Significant structural changes and inflammatory infiltration |
| 8-12 | Severe | Diffuse ulceration, crypt loss, and deep inflammation |

**
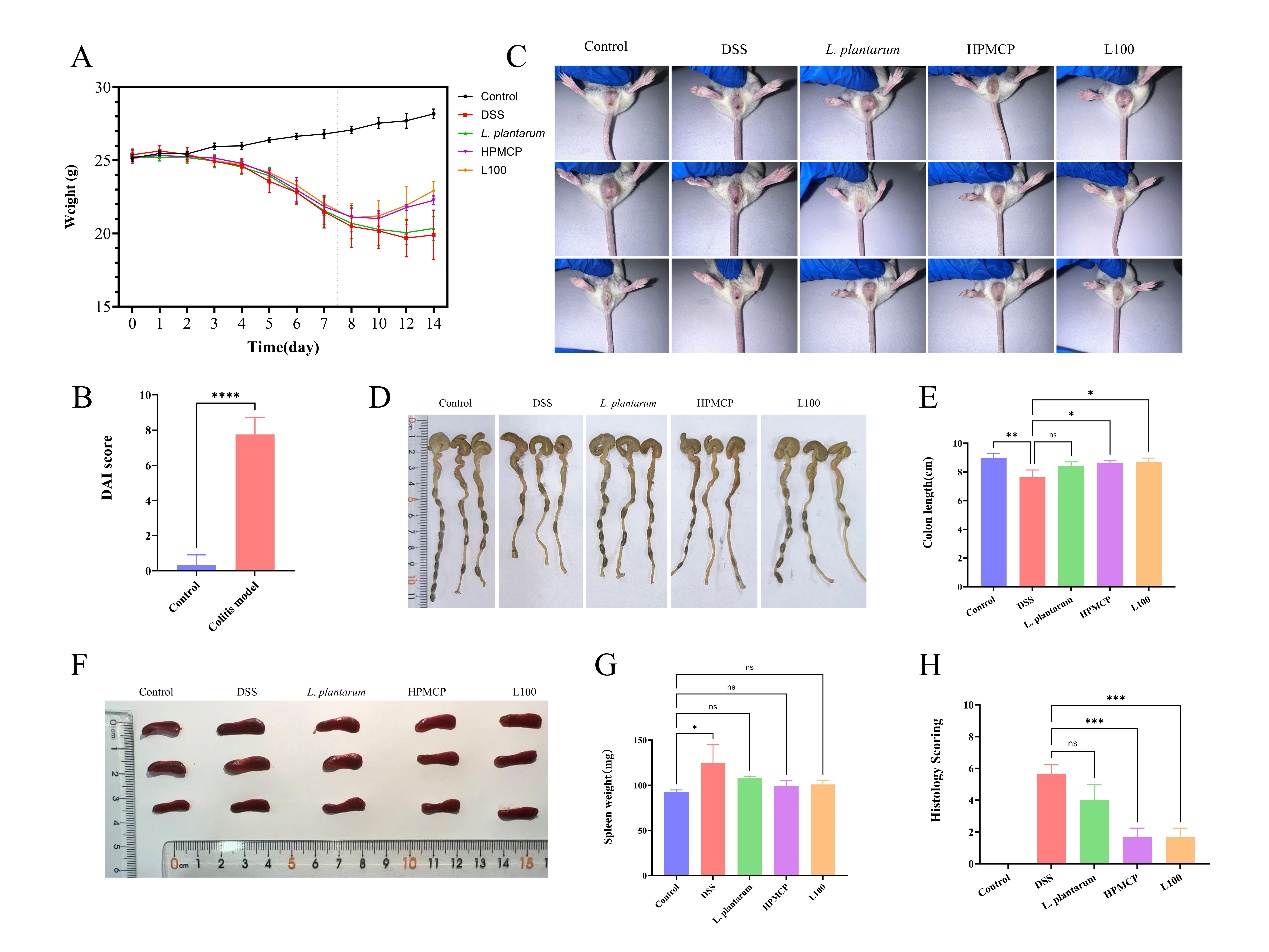
**

**Supplementary Figure S1**

**A：Changes in body weight of mice during the experimental period.**

**During the establishment of the DSS-induced acute colitis model, body weight loss can be used as one of the reference indicators for successful model induction. Meanwhile, monitoring body weight changes during the treatment period after modeling can preliminarily reflect the recovery status of the mice.**

**B：Disease Activity Index (DAI) score. (p < 0.05, *p < 0.01, **p < 0.001, ***p < 0.0001)**

**The Disease Activity Index (DAI) score is a macroscopic functional parameter used to comprehensively evaluate the severity and progression of colitis. Compared with the control group, mice in the DSS-induced colitis model group exhibited significantly elevated DAI scores. The scores predominantly ranged between 6 and 8 (detailed scoring criteria and severity grading are shown in Tables 1 and 2), with most animals presenting moderate disease activity. These results preliminarily indicate that DSS treatment successfully induced an acute colitis model.**

**C：Photographs of rectal bleeding in mice from different groups during the treatment period.**

**During DSS-induced model establishment, mice in the model group exhibited severe colitis-associated symptoms, including obvious rectal bleeding and diarrhea. During the subsequent treatment phase, rectal bleeding was barely detectable in mice treated with HPMCP granules and L100 granules. In the L. plantarum group, rectal bleeding was largely resolved; however, mild perianal erythema remained observable in some mice. In contrast, rectal bleeding in the DSS group showed little improvement, with all mice displaying pronounced perianal hemorrhage and redness.**

**These observations preliminarily suggest that HPMCP granules and L100 granules accelerate the alleviation of acute colitis symptoms in mice. Visual assessment of rectal bleeding can therefore serve as an auxiliary indicator for evaluating therapeutic efficacy and determining experimental endpoints.**

**D：Representative photographs of the cecum and colon from mice in different groups (n = 3).**

**E:** **Colon length of mice in different groups (n = 3).**

**Colon shortening is considered an indirect indicator of colonic inflammation. Mice in the DSS group exhibited a marked reduction in colon length (approximately 7.63 cm). In contrast, treatment with HPMCP sustained-release granules and L100 sustained-release granules significantly alleviated DSS-induced colon shortening.**

**F:** **Representative photographs of dissected spleen tissues from mice in different groups (n = 3).**

**G：Spleen weight of mice in different groups (n = 3).**

**Splenomegaly is another manifestation of DSS-induced colitis. Treatment with HPMCP sustained-release granules and L100 sustained-release granules effectively alleviated DSS-induced spleen enlargement.**

**H：Histology scores of colonic tissues in mice from different groups (n = 3).**

**Histological scoring was performed based on a 0–12 scale (Tables 3 and 4). Mice in the DSS group exhibited scores in the range of 6–7, indicating moderate severity. Mice in the L. plantarum group, which received oral administration of L. plantarum without protective materials, showed only modest recovery compared with the DSS group. In contrast, treatment with HPMCP and L100 sustained-release granules resulted in significantly improved histology scores, approaching those of the control group and outperforming the L. plantarum group. These results preliminarily indicate that the sustained-release granules prepared in this study can effectively alleviate colitis.**

**Data are presented as means ±SD. Not significant (ns)P≥0.05, *P<0.05, **P<0.01, and***p<0.001.**

**
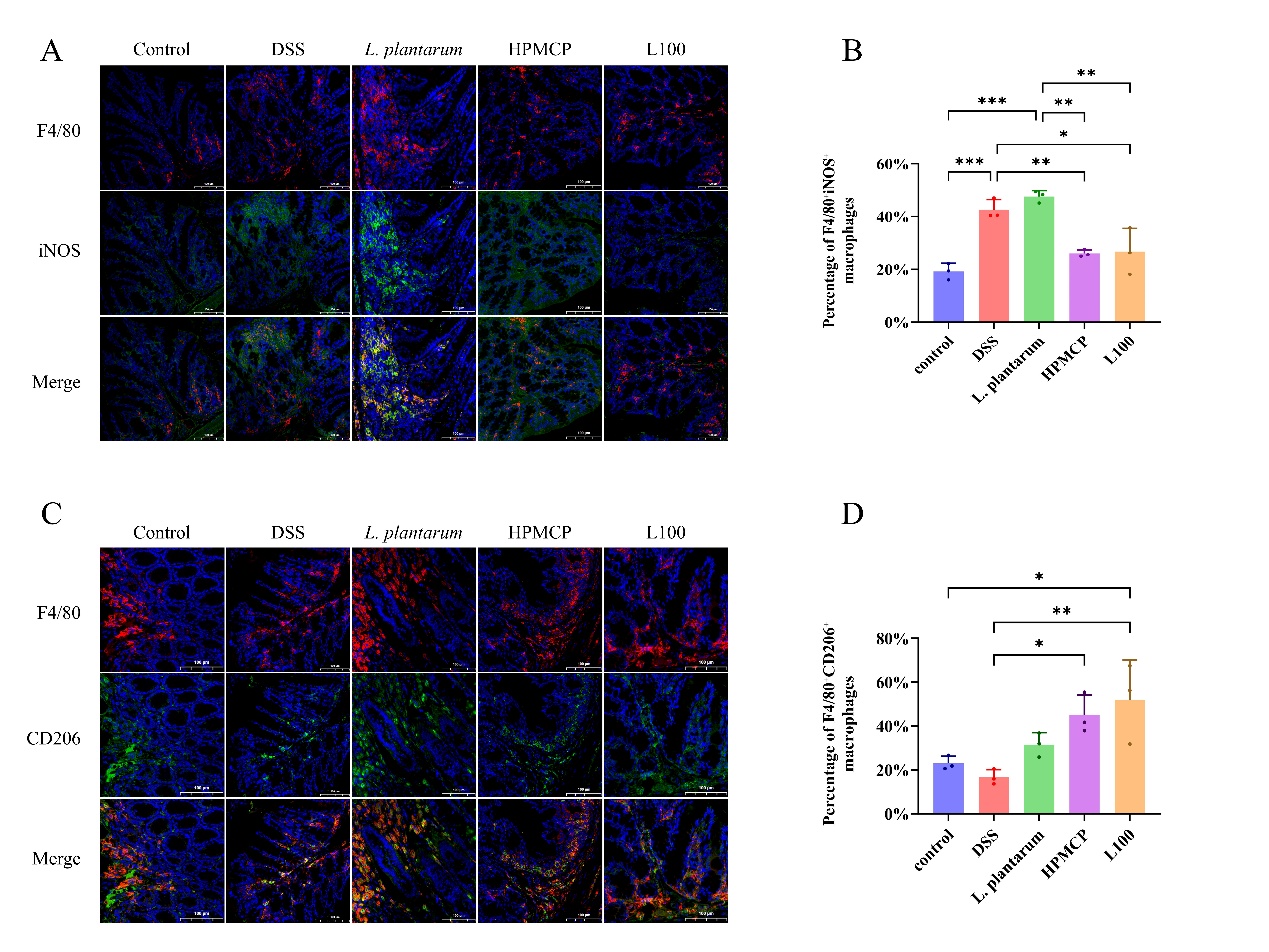
**

**Supplemental Figure S2**

**A: Representative immunofluorescence images showing co-localization of F4/80 and iNOS, markers of M1 macrophages.**

**B: Percentage of F4/80⁺iNOS⁺ macrophages in colonic tissues.**

**C: Representative immunofluorescence images showing co-localization of F4/80 and CD206, markers of M2 macrophages.**

**D: Percentage of F4/80⁺CD206⁺ macrophages in colonic tissues.**

**Data are presented as means ±SD. Not significant (ns)P≥0.05, *P<0.05, **P<0.01, and***p<0.001.**

**Immunofluorescence staining was performed to simultaneously label F4/80+iNOS (M1) and F4/80+CD206 (M2) macrophages, in order to assess the effects of the two sustained-release granules on M1 and M2 macrophage populations in colonic tissues. As shown in Figure S2, treatment with HPMCP and L100 granules significantly reduced the expression of F4/80+iNOS compared with the DSS group, indicating a decrease in M1 macrophages. Meanwhile, both granules markedly increased F4/80+CD206 expression, reflecting an increase in M2 macrophages. These results suggest that HPMCP and L100 granules modulate the M1/M2 macrophage polarization balance, particularly promoting M2 polarization.**
