# Supplementary figures and images for: Sustained-release enteric formulations of Lactiplantibacillus plantarum based on granulation technology: preparation and therapeutic evaluation in acute colitis
Source: BMC Biotechnol. 2026 Apr 21;26:73. doi: 10.1186/s12896-026-01157-7 (PMC13231606; doi:10.1186/s12896-026-01157-7)

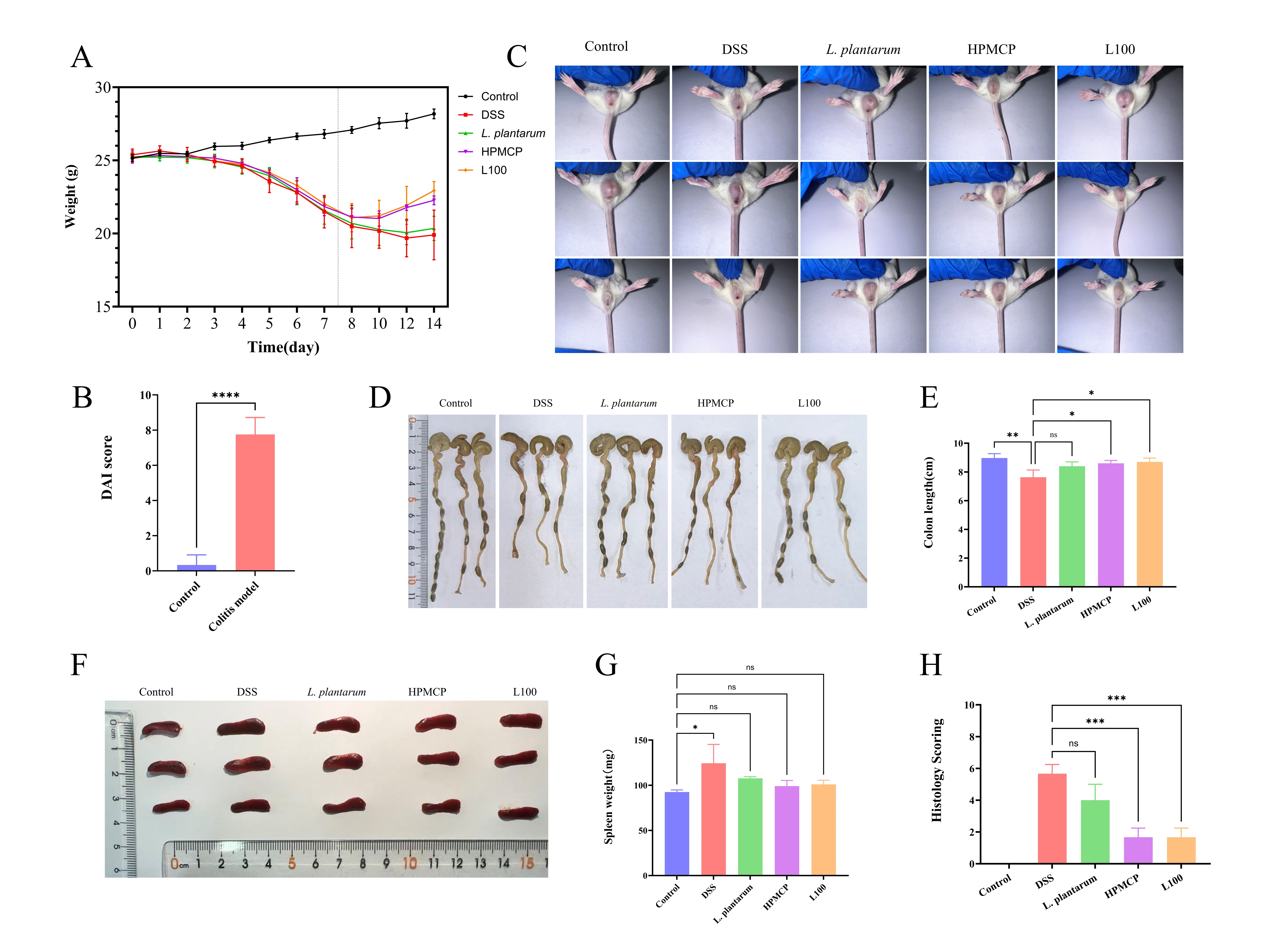

Supplement: Supplementary file 1 — Supplementary material 1 [file 12896_2026_1157_MOESM1_ESM.zip › 12896_2026_1157_MOESM1_ESM/Supplementary Figures/Supplementary Figure S1/Supplementary Figure S1.jpeg]

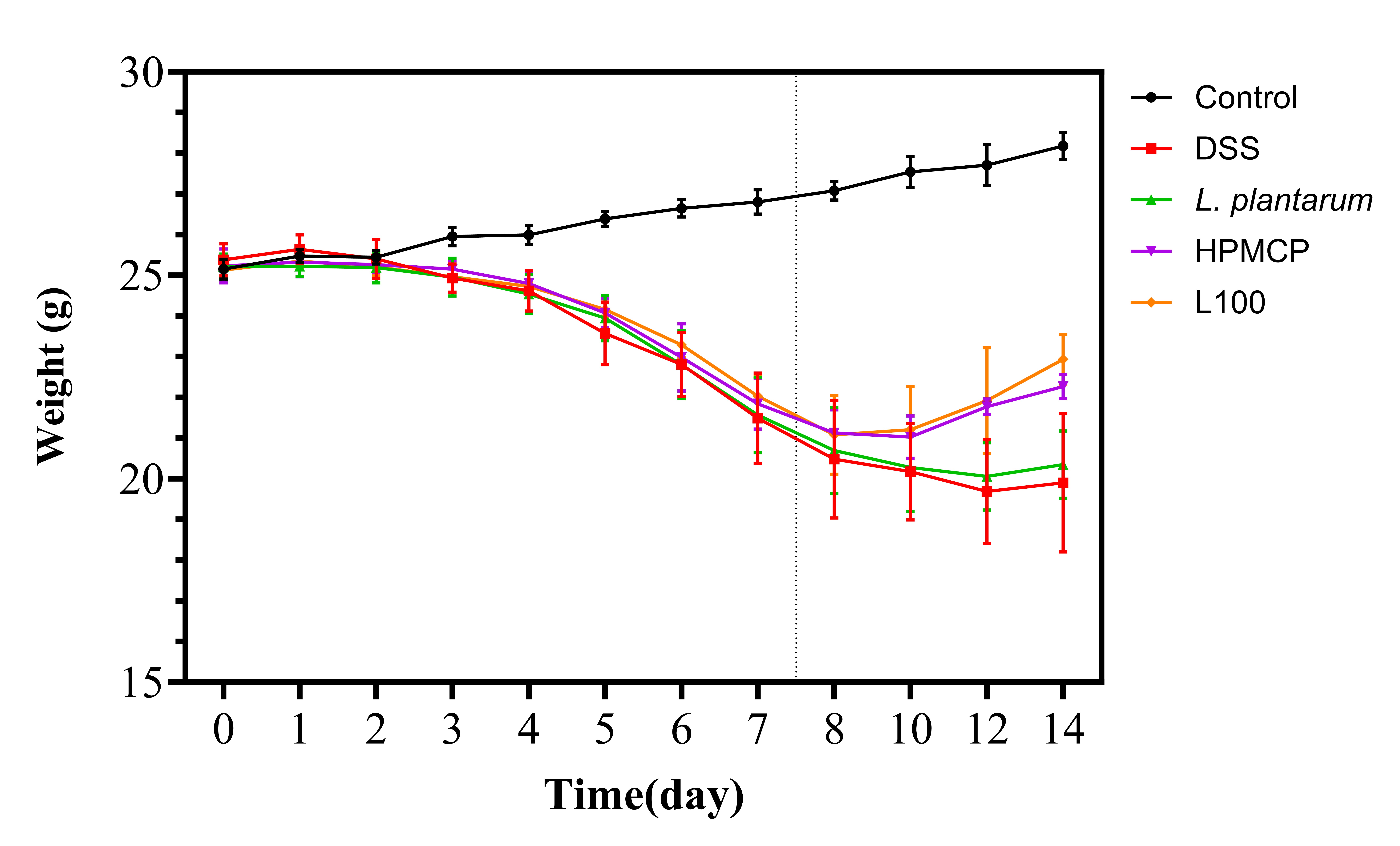

Supplement: Supplementary file 1 — Supplementary material 1 [file 12896_2026_1157_MOESM1_ESM.zip › 12896_2026_1157_MOESM1_ESM/Supplementary Figures/Supplementary Figure S1/Supplementary Figure S1A.jpeg]

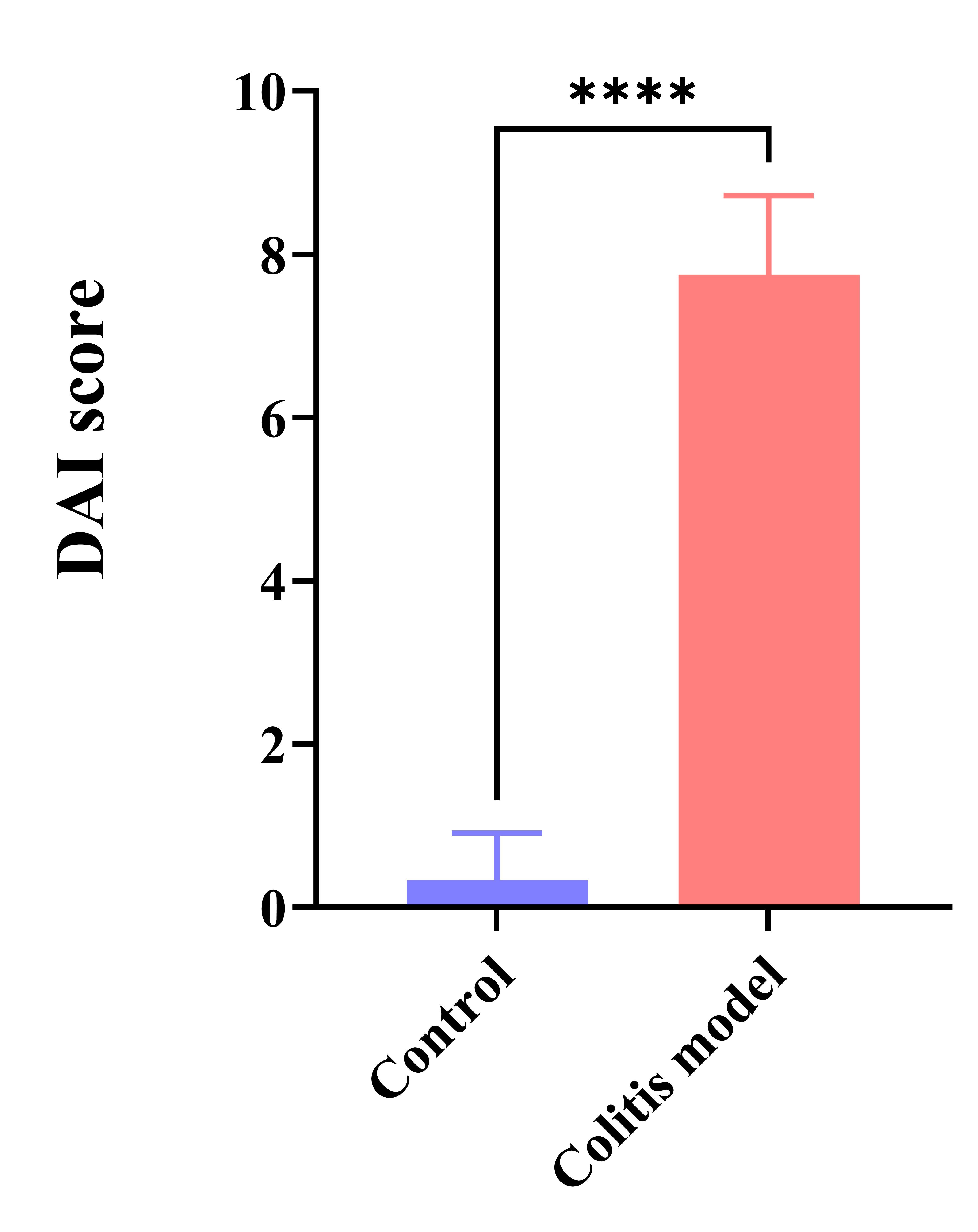

Supplement: Supplementary file 1 — Supplementary material 1 [file 12896_2026_1157_MOESM1_ESM.zip › 12896_2026_1157_MOESM1_ESM/Supplementary Figures/Supplementary Figure S1/Supplementary Figure S1B.jpeg]

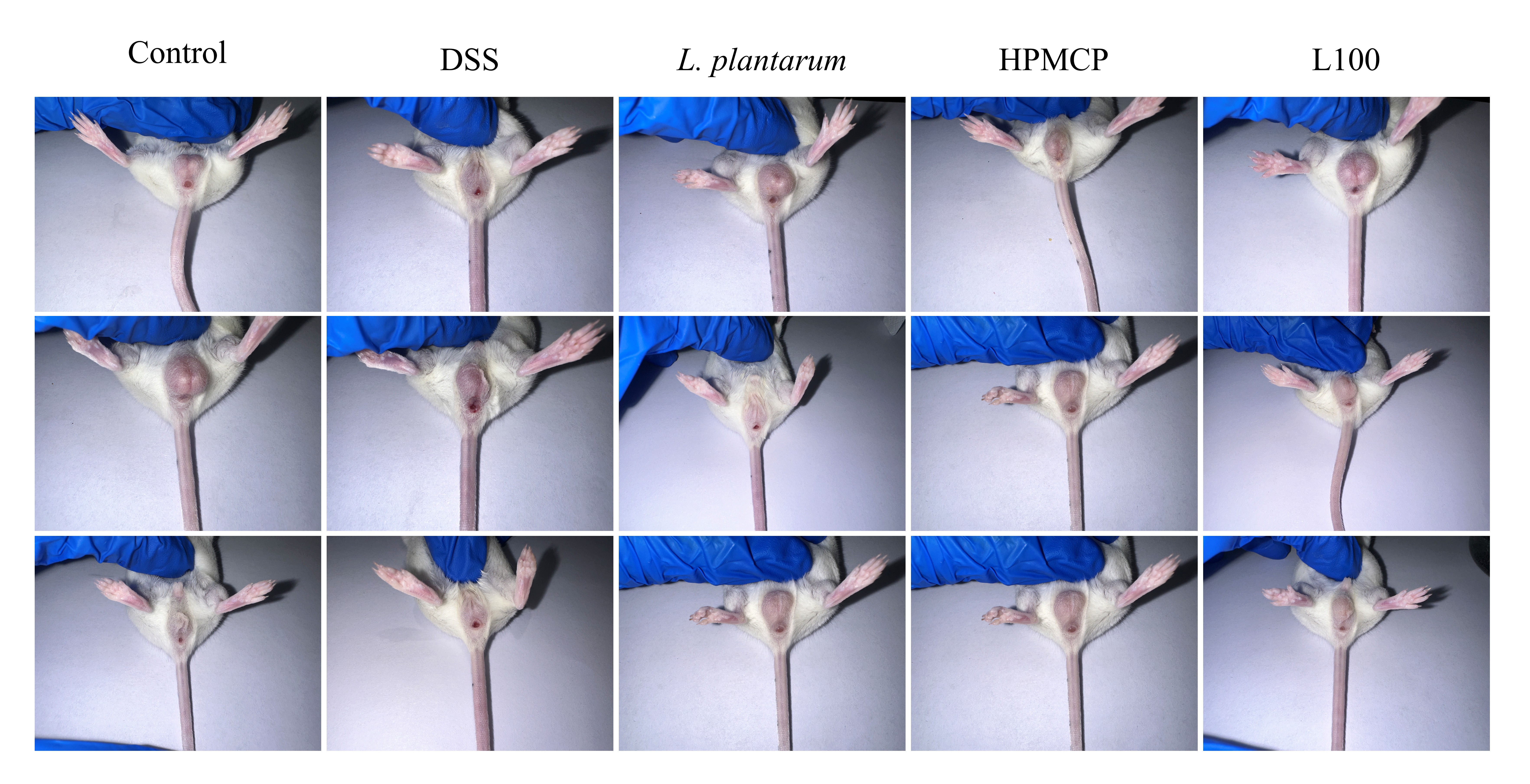

Supplement: Supplementary file 1 — Supplementary material 1 [file 12896_2026_1157_MOESM1_ESM.zip › 12896_2026_1157_MOESM1_ESM/Supplementary Figures/Supplementary Figure S1/Supplementary Figure S1C.jpeg]

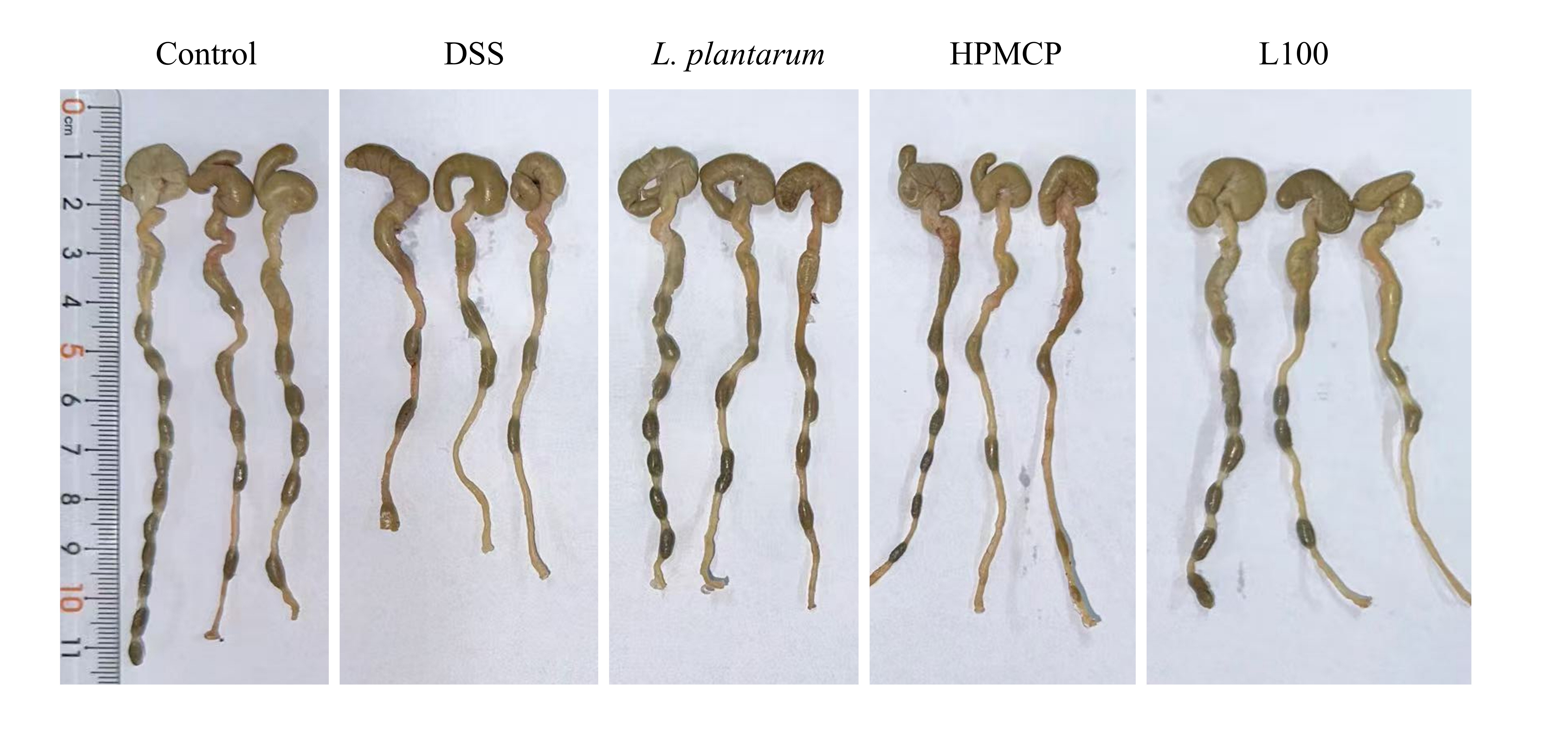

Supplement: Supplementary file 1 — Supplementary material 1 [file 12896_2026_1157_MOESM1_ESM.zip › 12896_2026_1157_MOESM1_ESM/Supplementary Figures/Supplementary Figure S1/Supplementary Figure S1D.jpeg]

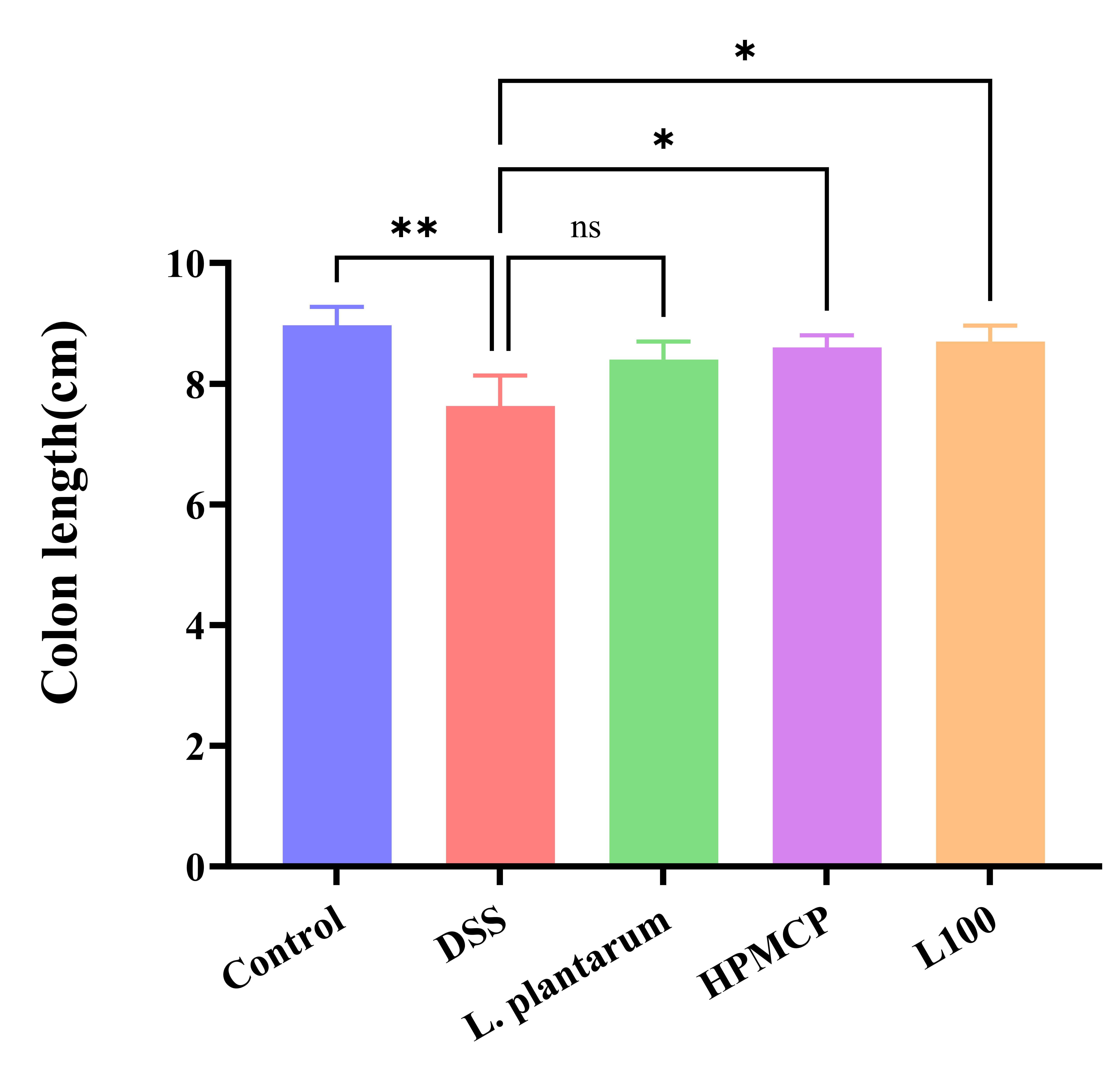

Supplement: Supplementary file 1 — Supplementary material 1 [file 12896_2026_1157_MOESM1_ESM.zip › 12896_2026_1157_MOESM1_ESM/Supplementary Figures/Supplementary Figure S1/Supplementary Figure S1E.jpeg]

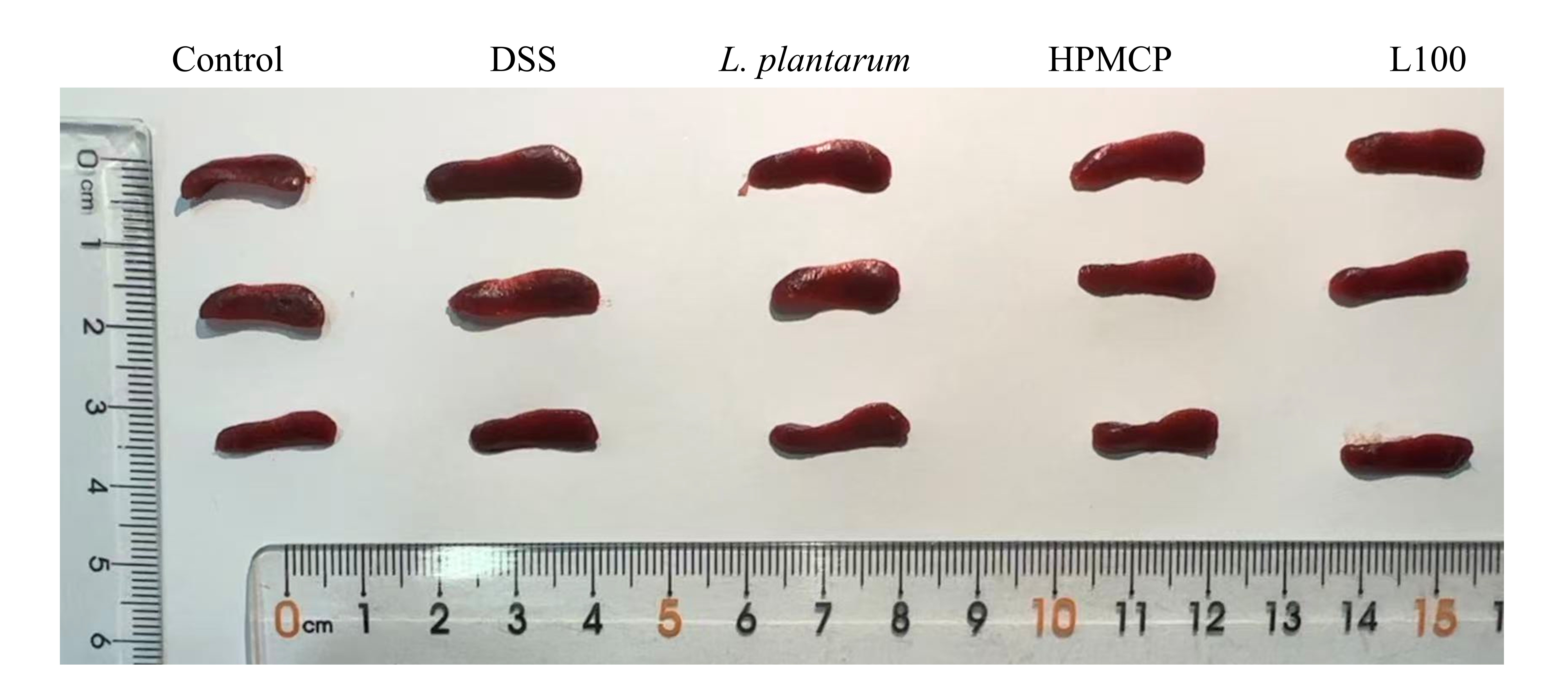

Supplement: Supplementary file 1 — Supplementary material 1 [file 12896_2026_1157_MOESM1_ESM.zip › 12896_2026_1157_MOESM1_ESM/Supplementary Figures/Supplementary Figure S1/Supplementary Figure S1F.jpeg]

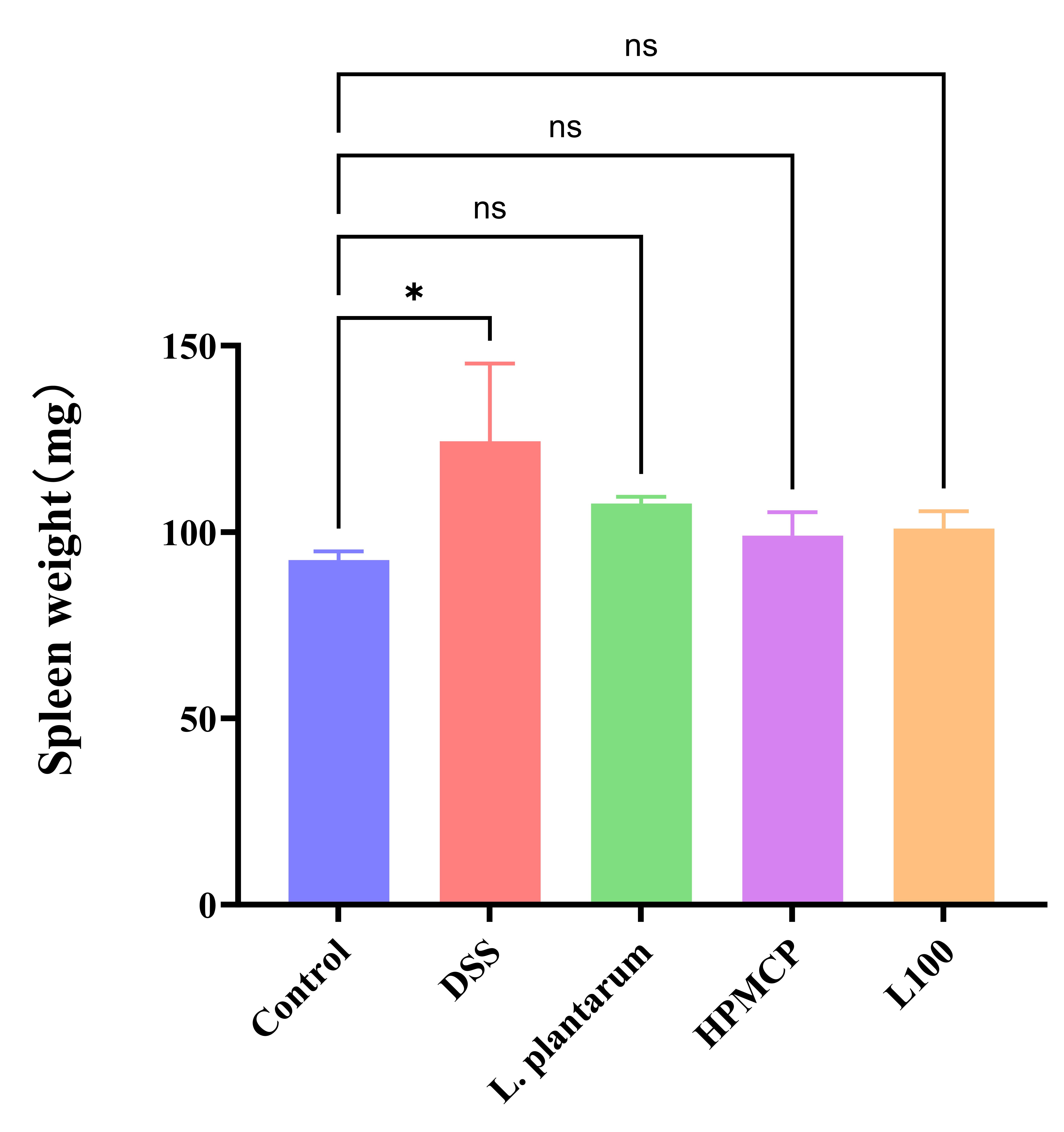

Supplement: Supplementary file 1 — Supplementary material 1 [file 12896_2026_1157_MOESM1_ESM.zip › 12896_2026_1157_MOESM1_ESM/Supplementary Figures/Supplementary Figure S1/Supplementary Figure S1G.jpeg]

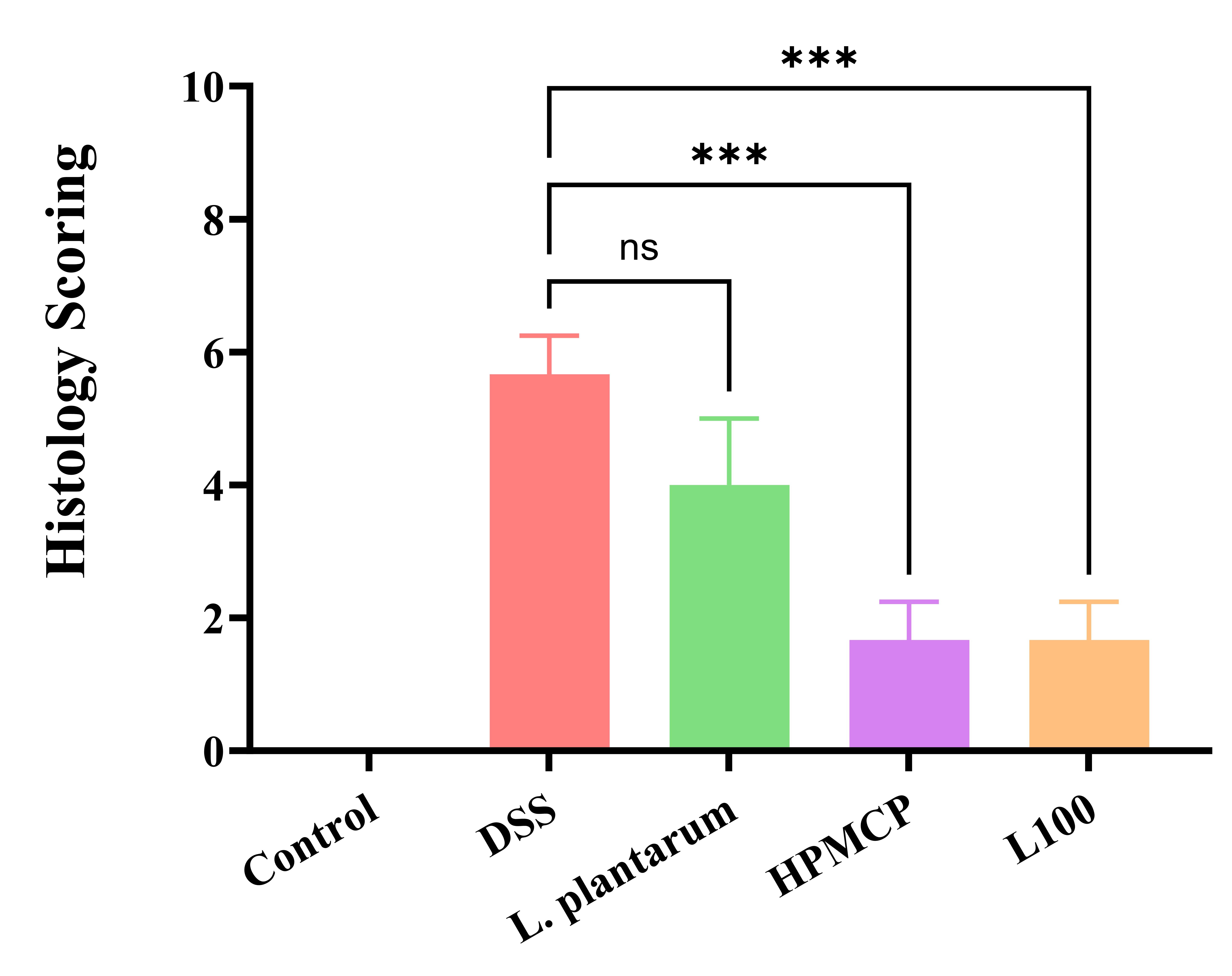

Supplement: Supplementary file 1 — Supplementary material 1 [file 12896_2026_1157_MOESM1_ESM.zip › 12896_2026_1157_MOESM1_ESM/Supplementary Figures/Supplementary Figure S1/Supplementary Figure S1H.jpeg]

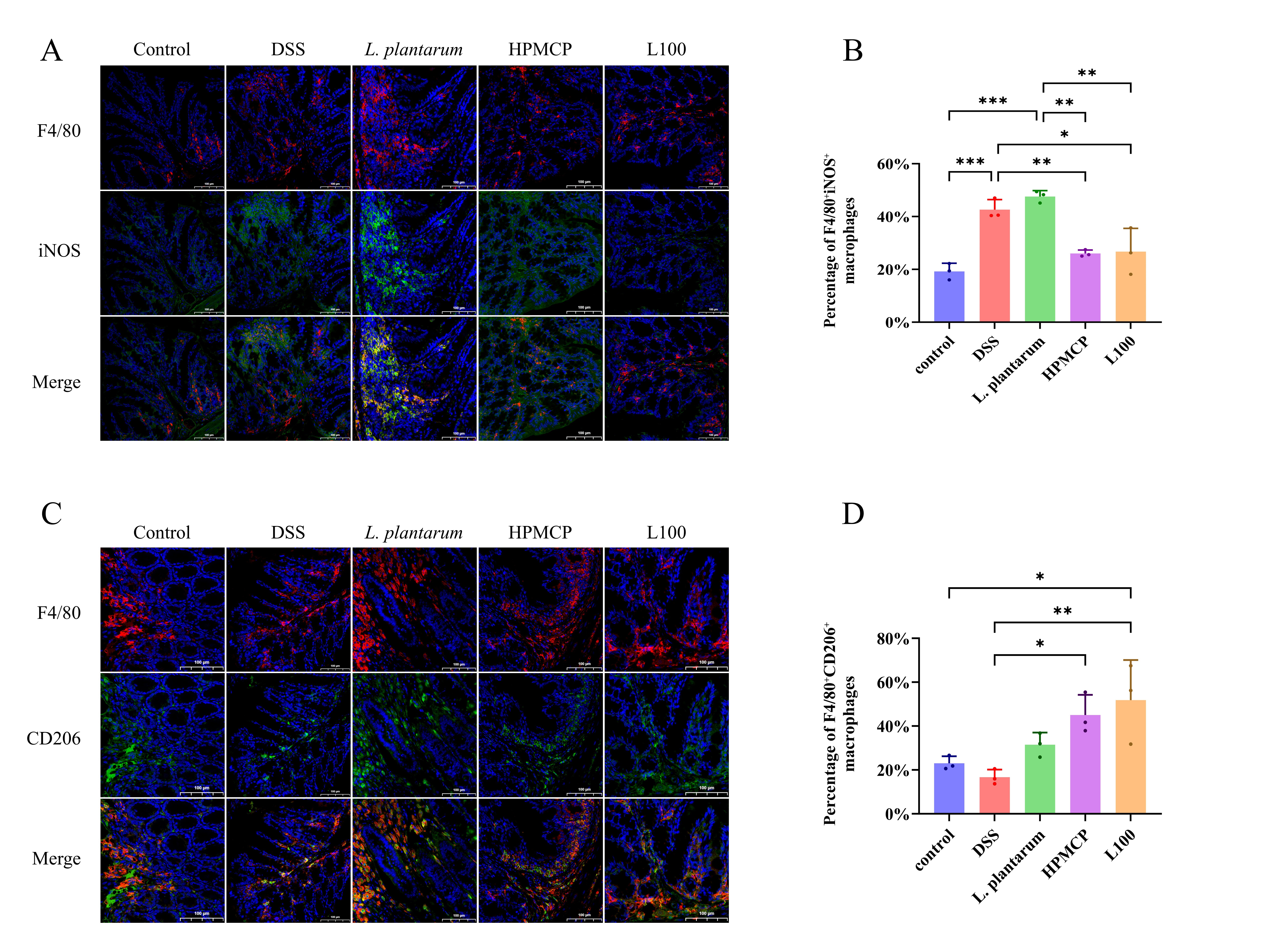

Supplement: Supplementary file 1 — Supplementary material 1 [file 12896_2026_1157_MOESM1_ESM.zip › 12896_2026_1157_MOESM1_ESM/Supplementary Figures/Supplementary Figure S2/Supplementary Figure S2.jpeg]

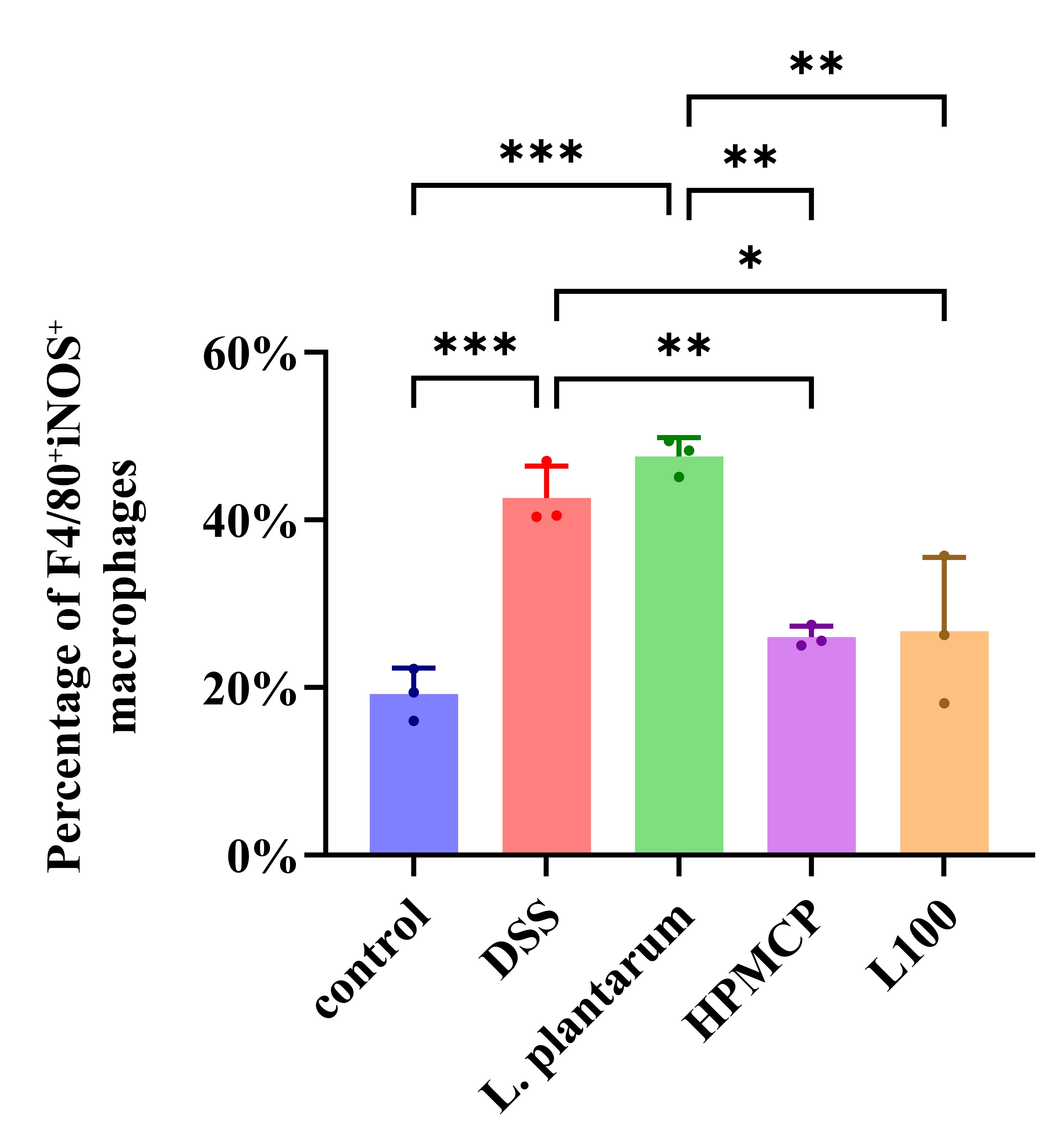

Supplement: Supplementary file 1 — Supplementary material 1 [file 12896_2026_1157_MOESM1_ESM.zip › 12896_2026_1157_MOESM1_ESM/Supplementary Figures/Supplementary Figure S2/Supplementary Figure S2B.jpeg]

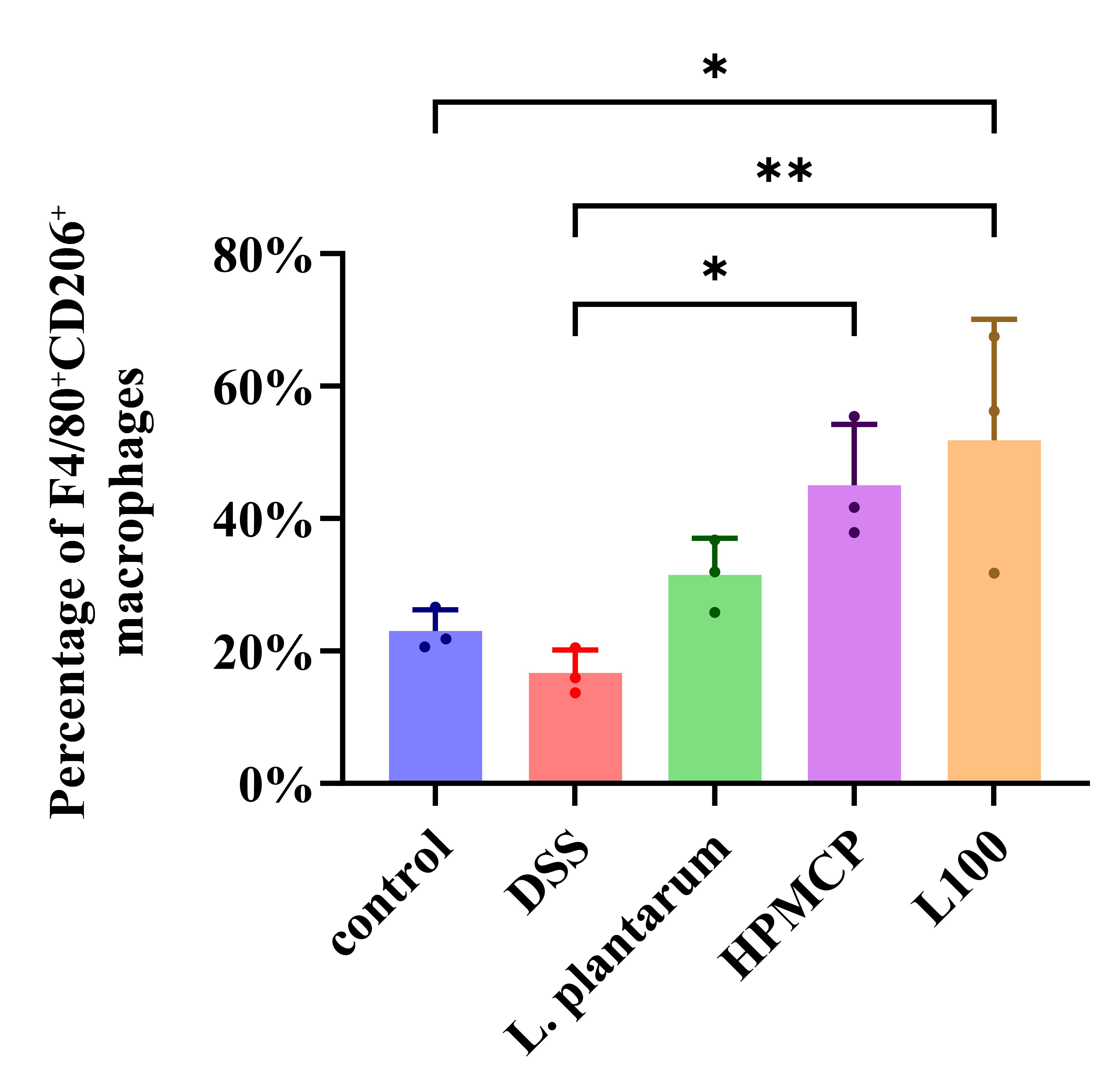

Supplement: Supplementary file 1 — Supplementary material 1 [file 12896_2026_1157_MOESM1_ESM.zip › 12896_2026_1157_MOESM1_ESM/Supplementary Figures/Supplementary Figure S2/Supplementary Figure S2D.jpeg]
